# A DoE-Dual centrifugation-driven screening platform for liposomal incorporation of poorly soluble APIs: Application to the PROTAC ACBI2

**DOI:** 10.1016/j.ijpx.2026.100627

**Published:** 2026-08-01

**Authors:** Miriam Jaki, Monika Köll-Weber, Jan Lembeck, Maximilian Wittmann, Regine Süss

**Affiliations:** a University of Freiburg, Institute of Pharmaceutical Sciences, Department of Pharmaceutics, Sonnenstraße 5, 79104 Freiburg, Germany; b University of Freiburg, Institute of Pharmaceutical Sciences, Department of Pharmaceutics, Hermann-Herder-Str. 9, 79104 Freiburg, Germany; c Boehringer Ingelheim Pharma GmbH & Co. KG, Birkendorfer Straße 65, 88400 Biberach/Riss, Germany

**Keywords:** Design of experiments, HPLC, PROTACs, Liposomes, Dual centrifugation

## Abstract

A fast and efficient screening platform is presented for formulating poorly soluble drugs, such as proteolysis-targeting chimeras (PROTACs), into liposomes. Driven by Design of Experiments (DoE) and powered by dual centrifugation, this platform enables high-throughput parallel preparation alongside a newly validated, single-run HPLC method for the simultaneous quantification of all lipid components and hydrophobic payloads. Extended applicability of this HPLC method is demonstrated across three distinct PROTACs and diverse lipid matrices.

Mechanistic protocol implementation using the SMARCA2-degrader ACBI2 reveals that the anionic phospholipid DOPG drastically enhances incorporation, whereas cholesterol hinders it due to electrostatic forces and bilayer flexibility demands. Notably, biophysical analysis shows that ACBI2 modulates bilayers in a cholesterol-like manner, increasing lipid packing order in the liquid-crystalline phase while reducing it in the gel phase. Thus, cholesterol-induced packing density directly impairs drug loading.

Guided by the DoE model, four uniform lead ACBI2 formulations were identified, achieving robust drug concentrations of up to 2.3 mM, which corresponds to a drug-to-lipid ratio of 5.5–6.0% (*n/n*). Finally, all lead candidates were successfully validated *in vitro*, demonstrating activity comparable to that of the DMSO controls.

The successful implementation of the presented platform for ACBI2 confirms this methodology as a powerful, predictable workflow for engineering challenging lipid-based nanocarriers.

## Highlights

• Mixture-process design for efficient formulation screening of PROTAC-liposomes.
• RP-HPLC-DAD-CAD method for simultaneous quantification of PROTACs and liposomal components.
• ACBI2 incorporation follows an inverse relationship of DOPG and cholesterol content.
• ACBI2 interacts cholesterol-like with phospholipid bilayers.
• Development of PROTAC-liposomes with high drug loads.

## Graphical abstract

**Figure:**
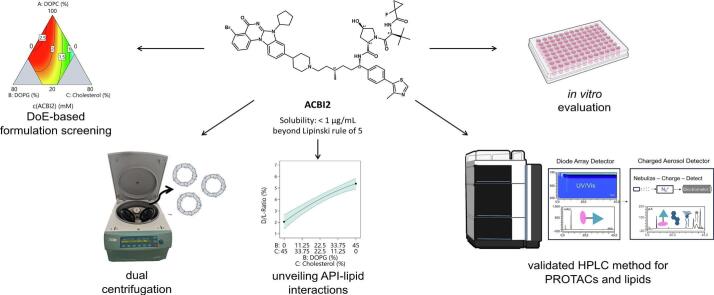

## 1 Introduction

PROTACs are a novel, emerging class of molecules that can selectively degrade target proteins *via* the ubiquitin-proteasome system. In order to do so, PROTACs bind to the target protein and an E3 ligase, and this resulting proximity induces ubiquitination and subsequent degradation of the target protein by the proteasome (Békés et al., 2022; Gao et al., 2020; Liu et al., 2022).

This approach offers major potential because it enables the targeting of proteins that have been previously considered undruggable, as a PROTAC does not require an enzymatic activity of the target protein to be either present or targeted to be effective. PROTACs only require a binding site. It is estimated that only approximately 20% of known protein targets can be addressed using classic small-molecule inhibitors, and PROTACs may notably increase this number (Békés et al., 2022; Gao et al., 2020). Many PROTACs are currently in clinical trials, showing encouraging results, and the first PROTAC, vepdegestrant, has recently received FDA approval (Amalraj and Parthasarathy, 2025; Arenas-Moreira et al., 2026; FDA, 2026; M et al., 2025).

Despite these promising perspectives, PROTACs face major drawbacks: due to their mode of action, they are heterobifunctional, which renders them generally large (∼1 kDa), polar, and beyond Lipinski's rule of five (Ro5) (Atilaw et al., 2021; Békés et al., 2022; Gao et al., 2020; Lipinski et al., 2001). Consequently, most PROTACs have poor aqueous solubility, poor membrane permeability, and therefore low oral bioavailability (Chen et al., 2022; Garber, 2022; Liu et al., 2022). Key characteristics for the prediction of bioavailability of the PROTAC ACBI2 in comparison to other poorly soluble Ro5 and beyond Ro5 drugs are provided in the Supplementary Information (Table A.1).

Parenteral application can be used to enhance bioavailability. Incorporating drugs into nanocarriers can further improve tumor accumulation *via* the enhanced permeability and retention effect and decrease on-target-off-tumor effects (Matsumura and Maeda, 1986). Thus, incorporating PROTACs into nanocarriers is an approach that is currently being investigated (Chen et al., 2022; Cimas et al., 2020; Fu et al., 2021; Jiang et al., 2023; Kou et al., 2023; Pan et al., 2025; Saraswat et al., 2022; Vartak et al., 2022). Among other nanocarriers, liposomes are the most established type to date, as indicated by the highest number of drug approvals by the FDA, and their amphiphilic properties hold potential for PROTAC incorporation (Wang et al., 2022). Although PROTACs have been incorporated into liposomes in previous studies, to our knowledge, none of them provide detailed information on formulation development and improvement. Furthermore only maximum drug loads of approximately 4.2% (*n/n*) could be reached, with most formulations incorporating less than 2% (*n*_*drug*_*/n*_*lipids*_) (Arenas-Moreira et al., 2025; Chen et al., 2025; Fu et al., 2021, Fu et al., 2020; Kou et al., 2023; Patel et al., 2025; Saraswat et al., 2022, Saraswat et al., 2020; Wang et al., 2024; Wen et al., 2026; Zhang et al., 2025). Therefore, we believe that this delivery vehicle has not reached its full potential for PROTACs yet.

In this work, we present approaches for the development and optimization of nanoparticulate drug delivery systems containing PROTACs. We established a screening approach that provides maximum information about the formulation composition and process parameters while minimizing the required amount of active pharmaceutical ingredient (API). This is particularly noteworthy given the synthetic complexity of PROTACs, which typically require multistep synthesis (Liu et al., 2022; Osman et al., 2025).

For this screening platform, we combined a DoE approach with dual centrifugation. DoE is a powerful statistical tool for planning and executing experiments. It enables the screening and optimization of processes with a reduced number of samples and provides more accurate and complete information than the one-factor-at-a-time (OFAT) approach (Beg, 2021; Lee, 2019).

Dual centrifugation is an *in-vial* homogenization technique that enables fast and material-saving preparation of liposomes and other nanocarriers with up to 40 formulations per batch (Koehler et al., 2023b; Massing et al., 2008). Lipid films are combined with low amounts of buffer and zircon oxide yttrium-stabilized beads and homogenized in a dual centrifuge before further hydration and dual centrifugation transforms them into liposomes (Bender et al., 2026; Koehler et al., 2023b; Massing et al., 2008). Downscaling capability of the process to lipid amounts as low as 1 mg has been demonstrated, making it a well-suited method for material saving and time-efficient formulation screening while keeping the drug load acceptably high (Bender et al., 2026).

The lamellarity of the liposomes prepared by dual centrifugation can also be adjusted. Low lipid concentrations of 20% during the first dual centrifugation step yield small unilamellar vesicles (SUVs), whereas a 60% lipid concentration results in small multilamellar vesicles (SMVs). A lipid concentration of 40% yields a mixture of both (Koehler et al., 2025, Koehler et al., 2023a).

The screening platform we developed combines screening of different lipid mixtures with screening of pH and lipid concentration during dual centrifugation. The pH was varied to investigate the influence of the charge from both the API and phospholipids on incorporation efficiency. Lipid concentration during dual centrifugation was incorporated into the DoE approach to investigate the effect of lamellarity on attributes such as incorporation efficiency, size, and polydispersity.

This platform approach was applied to the PROTAC ACBI2 to obtain liposomes with high drug loads and to elucidate its incorporation mechanism as a beyond Ro5 drug.

ACBI2 is a von-Hippel-Lindau (VHL)-based PROTAC that selectively degrades switch/sucrose non-fermentable (SWI/SNF)-related matrix-associated actin-dependent regulator of chromatin subfamily A, member 2 (SMARCA2), an ATPase subunit of the chromatin remodeling BRG1/BRM-associated factor (BAF) complex (SWI/SNF) (Kofink et al., 2022). SMARCA2 (BRM) and SMARCA4 (BRG1) are mutually exclusive ATPase subunits of this complex. In SMARCA4-deficient tumors, SMARCA2 can compensate for its loss, making selective SMARCA2 degradation by PROTACs an effective strategy to inhibit tumor growth (Cantley et al., 2022; Kofink et al., 2022). *SMARCA4* mutations are particularly prevalent in non-small cell lung cancer (NSCLC), and affected tumors are recognized as very aggressive with poor clinical outcomes (Armon et al., 2021; Dagogo-Jack et al., 2020). ACBI2 is insoluble in water and has very low oral bioavailability (Kofink et al., 2022). Therefore, incorporation into nanocarriers suitable for parenteral administration could potentially enhance its therapeutic applicability.

## 2 Materials & methods

### 2.1 Materials

ACBI1, ACBI2 and ACBI3 were kindly provided by Boehringer Ingelheim International GmbH (Ingelheim am Rhein, Germany) *via* its open innovation platform opnMe, available at https://www.opnme.com. 1,2-Dioleoyl-*sn*-glycero-3-phosphocholine (DOPC), 1,2-dioleoyl-*sn*-glycero-3-phospho-*rac*-glycerol (sodium salt) (DOPG), 1,2-distearoyl-*sn*-glycero-3-phosphoethanolamine-N-[methoxy(polyethylene glycol)-2000] (DSPE-mPEG_2000_) (sodium salt), 1,2-dioleoyl-*sn*-glycero-3-phosphoethanolamine (DOPE), 1,2-dioleoyl-3-trimethylammonium-propane (chloride salt) (DOTAP), 1,2-distearoyl-*sn*-glycero-3-phosphocholine (DSPC), 1,2-distearoyl-*sn*-glycero-3-phospho-*rac*-glycerol (sodium salt) (DSPG), and oleic acid were generously donated by Lipoid (Ludwigshafen, Germany). 1-stearoyl-2-hydroxy-*sn*-glycero-3-phosphocholine (18:0 LPC) and 1-oleoyl-2-hydroxy-*sn*-glycero-3-phosphocholine (18:1 LPC) were purchased from Avanti Polar Lipids LLC. (Alabaster, USA). Ammonium acetate (> 99%), laurdan, and cholesterol (≥ 99.5%) and were procured from Sigma-Aldrich (St. Louis, USA). D(+)-glucose (anhydrous) and ethanol (≥ 99.8%) were sourced from Fisher Scientific (Loughborough, UK). Methanol (HPLC ULTRA LC-MS grade, ≥ 99.9%) was supplied by VWR International (Leuven, Belgium). L-histidine (≥ 98.5%), sodium chloride (NaCl) (> 99.8%), D(+)-sucrose (≥ 99.5%), ammonia solution (25%), sulfuric acid (96%), dimethyl sulfoxide (DMSO) (> 99.5%), and acetic acid (100%) were obtained from Carl Roth GmbH & Co. KG (Karlsruhe, Germany). Sodium hydroxide (NaOH) was purchased from Avantor Performance Materials Poland S.A. (Gliwice, Poland), and hydrochloric acid (HCl) was acquired from Merck KgaA (Darmstadt, Germany). Purified water was obtained from an Arium Pro water purification system from Sartorius AG (Göttingen, Germany).

### 2.2 Methods

#### 2.2.1 Preparation of lipid-and-API-films

Stock solutions of ACBI2 and phospholipids were prepared using ethanol. These solutions were combined in a micro tube (Sarstedt AG & Co. KG, Nümbrecht, Germany) in the necessary proportions to manufacture vesicular phospholipid gels (VPGs) containing 50 mg of the desired molar ratios and API content. The ethanol mixture was then dried in a vacuum centrifuge (Concentrator plus, Eppendorf AG, Hamburg, Germany) at 30 °C for 3 h. The resulting dry lipid-and-API-films were stored at −20 °C until further use.

#### 2.2.2 Preparation of liposomes *via* dual centrifugation

The lipid-and-API-films were thawed in a desiccator. Then, 0.60 g of yttrium-stabilized zirconium oxide beads (1.4–1.6 mm) (Sigmund Lindner GmbH, Warmensteinach, Germany) were added to each vial, along with formulation buffer solution, to achieve the desired lipid concentration for dual centrifugation.

The vials were placed in a dual centrifuge (ZentriMix 380 R, Andreas Hettich GmbH & Co. KG, Tuttlingen, Germany) and homogenized at 2350 rpm and 10 °C for 30 min (unless otherwise specified). After adding additional buffer to the resulting VPGs, the vials were returned to the dual centrifuge and processed at 1500 rpm and 20 °C for 2 min (unless otherwise specified). The resulting liposomal dispersions had a lipid concentration of 50 mM.

The entire sample was then transferred to an Eppendorf tube and purified by centrifugation at 10,000 ×*g* and 22 °C for 10 min (Centrifuge 5417R; Eppendorf AG, Hamburg, Germany). The supernatant was transferred into a new vial to separate it from non-incorporated drug. The suitability of this purification method was confirmed using lipid-free samples (Table A.8).

Liposomal formulations for the DoE were produced in HiS buffer (50 mM histidine, 240 mM sucrose, pH 6.0) to warrant a stable pH during screening experiments. All other formulations were prepared in HiG buffer (20 mM histidine, 270 mM glucose, resulting pH 6.0 in formulations) to reduce buffer concentration considering market products and to enable cryoTEM imaging (Strickley and Lambert, 2021). A comparison of formulations prepared with either buffer is in Fig. A.10.

Liposome formulations containing DSPC and DSPG as the main lipids were preheated to 70 °C for 10 min using a Thermomixer comfort (Eppendorf AG, Hamburg, Germany) before processing in a dual centrifuge for 5 min at 2350 rpm and 40 °C to ensure a temperature above the phase transition of these lipids. After the addition of additional buffer, the sample was heated again to 70 °C for 5 min before the last dual centrifugation step, which was conducted as for all other liposome formulations. The impact of processing temperature and homogenization time on resulting DOPC/DOPG liposomes was investigated and the results are shown in Fig. A.11.

#### 2.2.3 Particle size and zeta potential

All measurements were performed using a Zetasizer Nano ZS (Malvern Panalytical GmbH, Kassel, Germany). For dynamic light scattering (DLS), a 6 μL sample was diluted with 1 mL of freshly filtered 25 mM NaCl- 250 mM sucrose medium (NaCl-sucrose medium) using 0.2 μm Chromafil® MV-20/25 cellulose mixed esters filters (Macherey-Nagel GmbH & Co. KG, Düren, Germany). Measurements were conducted in the 173° backscatter mode. Samples were equilibrated for 60 s at 25 °C and analyzed in triplicate at 25 °C, with each measurement consisting of ten individual measurements of 10 s. The viscosity of the dispersion medium was set to 1.1624 mPa s and its refractive index to 1.342. Intensity-weighted size distribution is displayed as *Z*-Average and size distribution as polydispersity index (PDI).

Zeta potential was measured using a Dip Cell (Malvern Panalytical GmbH, Kassel, Germany) and diluting 20 μL sample with 1 mL NaCl-sucrose medium (buffer composition shown above). Each sample was analyzed in triplicate, with 20 measurements per sample. Before and at the end of each measurement run accuracy was verified using a Zeta Potential Transfer Standard ZTA1240 (Malvern Panalytical GmbH, Kassel, Germany).

#### 2.2.4 Cryogenic transmission electron microscopy (cryoTEM) imaging of liposomes

The cryoTEM samples were prepared in 20 mM histidine and 270 mM glucose buffer. The samples were diluted to 10 mM lipid concentration. Subsequently, 4 μL of the sample were applied on a Quantifoil S 7/2 copper grid (Quantifoil, Jena, Germany) that was glow discharged by the PELCO easiGlow (Ted Pella, Redding, California, USA) in advance. Samples were blotted by the Vitrobot MK IV (ThermoFisher, Waltham, Massachusetts, USA) using the following parameters: blotting time of 3 s, blot force of 8, drain time of 2 s and chamber temperature of 22 °C with a relative humidity of 100%. Next, grids were vitrified by plunge freezing into liquid ethane and further stored in liquid nitrogen for cryogenic conditions. Images were taken with a FEI Talos L120C at 120 kV and a Ceta 16-megapixel camera (ThermoFisher, Waltham, Massachusetts, USA).

#### 2.2.5 Lipid-state observer (LISO)

A Lipid-State Observer (LISO) (Lipospec GmbH, Augsburg, Germany) was used for fluorescence-based analysis of the phase transition and lipid order of ACBI2-containing liposomes. The method was applied using Laurdan fluorescence, as introduced by Färber and Westerhausen (2022). The LISO measures the Generalized Polarization (*GP*) of a sample as a function of its temperature, thus allowing monitoring of the phase transition of a lipid bilayer. LISO enables the measurement of lipid phase transitions below the melting point of water, which is necessary for DOPC/DOPG liposomes (Färber and Westerhausen, 2022).

Samples were analyzed as follows: liposomes were diluted with 50 mM histidine 240 mM sucrose buffer, pH 6.0, to a lipid concentration of 3 mM and incubated with 0.5 mol-% Laurdan (Sigma-Aldrich, St. Louis, USA) in DMSO for 1 h at 600 rpm and 25 °C using a Thermomixer comfort (Eppendorf AG, Hamburg, Germany) followed by 12 h at 4–8 °C in the dark. 280 μL of the sample were transferred to a PCR tube (Sapphire, Greiner bio-one International GmbH, Frickenhausen, Germany) and inserted into the LISO together with a stirring bar (5 × 2 mm, PTFE; Carl Roth GmbH & Co KG, Karlsruhe, Germany). The measurement was performed after recording the background (HiS buffer) with the device set to “smart spectrum”, “simple freezing detection”, and a resolution of 0.3 K. The sample was stirred throughout the measurement.

DOPC/DOPG liposomes were preincubated for 10 min at 25 °C and subsequently cooled to −40 °C at a rate of 1 K/min. The temperature was then held for 10 min before being returned at 1 K/min to 25 °C. The upscan was utilized for analysis. Liposomes containing DSPC/DSPG as the main lipids were analyzed using a temperature ramp of 1 K/min from 20 to 70 °C after 10 min of pre-incubation. The sample was maintained at 70 °C for 10 min before being returned to 20 °C at a rate of 1 K/min. Downscans were used for the analysis.

#### 2.2.6 High performance liquid chromatography (HPLC)

A reversed-phase high-performance liquid chromatography (RP-HPLC) method was developed to quantify ACBI2, and other PROTACs, simultaneously with DOPC, DOPG, DSPE-mPEG_2000_, cholesterol, and degradation products. The method was validated in accordance with ICH guideline Q2(R2) regarding specificity, working range, precision, accuracy, detection limit, and quantitation limit (European Medicines Agency (EMA), 2023).

The drug-to-lipid ratio (*D/L ratio*) and incorporation efficiency (*IE*) were determined to characterize the resulting liposomes.

(1) $$D/L\;\mathrm{ratio}=\frac{c{\left(\mathrm{ACBI}2\right)}_{\mathrm{incorporated}}}{c\left(\mathrm{lipids}\right)}*100$$

(2) $$\mathrm{IE}=\frac{c{\left(\mathrm{ACBI}2\right)}_{\mathrm{incorporated}}}{c{\left(\mathrm{ACBI}2\right)}_{\mathrm{theoretic}}}*100$$

where *c(ACBI2)*_*incorporated*_ = concentration of incorporated ACBI2, *c(lipids)* = total concentration of lipids in the formulation, and *c(ACBI2)*_*theoretic*_ = theoretical concentration of ACBI2 at 100% incorporation.

Given that ACBI2 has no detectable aqueous solubility, all non-entrapped ACBI2 precipitated non-homogeneously and hindered accurate 100% measurements (Kofink et al., 2022). For this reason, IE was calculated using a theoretical 100% value.

##### 2.2.6.1 Preparation of eluents for HPLC

The eluents were freshly prepared prior to use. Eluent A (4 mM ammonium acetate in water, pH 4.0) was prepared by dissolving the required amount of ammonium acetate in purified water, followed by pH adjustment with 6 M acetic acid. For eluent B (4 mM ammonium acetate in methanol), ammonium acetate was dissolved in methanol and ultrasonicated for 1 min.

##### 2.2.6.2 HPLC method

Samples for HPLC analysis were processed using an UltiMate 3000 HPLC System (Thermo Fisher Scientific GmbH, Dreieich, Germany). This system consisted of a quaternary pump (LPG-3400SD), an autosampler (WPS-3000TSL), a column compartment (TCC-3000SD), a diode array detector (DAD) (DAD-3000), and a charged aerosol detector (CAD) (Dionex Corona CAD; Dionex Softron GmbH, Germering, Germany). The flow rate was maintained at 1 mL/min, and the autosampler temperature was set to 20 °C. Each sample was injected at a volume of 10 μL. Separation was achieved at 45 °C on a reversed-phase Luna C18(2) column (150 mm × 4.6 mm) with a 3 μm particle size, supplied by Phenomenex Ltd. (Aschaffenburg, Germany). The method was initialized with a 5 min equilibration step at 80% A and 20% B prior to sample injection. Following the injection, a gradient was applied to 80% B in 4 min, with an increase to 92% B at 4.5 min. This condition was maintained for 27.5 min. At 32 min, B was increased to 97% within 0.2 min and maintained at this level for 11.3 min before the pump returned to the starting conditions (20% B) within 1.5 min.

All APIs were quantified using a diode array detector on separate absorption channels, each with a bandwidth of 2 nm and a reference bandwidth of 10 nm. ACBI1, ACBI2, and ACBI3 were detected at 263, 242, and 249 nm, respectively.

The charged aerosol detector used for lipid quantification was set to an inlet pressure of 35 psi, with an evaporation temperature factory fixed at 30 °C, a data acquisition rate of 10 Hz, and a maximum detection threshold of 500 pA. The HPLC system was equilibrated with two blank runs. Data was analyzed using Chromeleon® software 7.2.10 ES (Thermo Fisher Scientific, Dreieich, Germany).

HPLC samples were prepared by diluting samples 1:20 up to 1:30 in ethanol and thorough homogenization. All samples were analyzed in triplicate. For more details and validation results, see Supporting Information.

#### 2.2.7 Design of experiments (DoE)

The DoE was carried out using Stat-Ease 360 software (23.1.9) (Stat-Ease, Inc., Minneapolis, USA). We selected a lipid mixture consisting of DOPC, DOPG, cholesterol, and DSPE-mPEG_2000_ for the experiments. In mixture experiments, it is necessary to optimize all ingredients simultaneously, as the outcome usually depends on the relative proportion of each component, not on individual amounts. Therefore, fractional-factorial designs or classical response-surface models are neither effective in theory, nor in practice (Abdel-Hafez et al., 2014; Beg, 2021).

As it was not reasonable to include the full range of each component, particularly cholesterol and DSPE-mPEG_2000_, an optimal design was chosen over a simplex design, with the constraints shown in Table 1 (Beg, 2021; Kowalski et al., 2000). D-optimality was chosen to reduce the generalized variance of the estimated coefficients and therefore to get low standard errors across the parameters, making it well-suited for screening purposes (Beg, 2021; Lee, 2019).

**Table 1:** Mixture components and process factors investigated in the DoE, including the design space of the experimental plan.

Component	Type	Name	Units	Minimum	Maximum	Levels
A	Mixture	DOPC	%	20	100	
B	Mixture	DOPG	%	0	50	
C	Mixture	Cholesterol	%	0	45	
D	Mixture	DSPE-mPEG_2000_	%	0	5	
E	Process	pH		5	7	5, 6, 7
F	Process	c(lipid)	%	20	60	20, 40, 60

The process parameters investigated in the experiment were pH and lipid concentration during dual centrifugation (Table 1). To minimize stability issues related to the phospholipids, the pH-range was restricted to 5–7, using discrete values (Duché and Sanderson, 2024; Grit et al., 1989; Zuidam and Crommelin, 1995).

Simultaneously obtaining information on mixture and process parameters typically requires a large number of runs, as each mixture parameter interaction must be crossed with each process parameter. However, Kowalski-Cornell-Vining (KCV) models drastically reduce the required number of experimental runs by eliminating less relevant interactions (Kowalski et al., 2000). In these models, mixture model interactions, as well as pure quadratic and two-factor interactions of the process variables, are assessed. The interaction between the mixture and process parameters is restricted to the most critical ones: those between the linear mixture terms and the main effect terms of the process variables (Kowalski et al., 2000).

The resulting model for our D-optimal KCV design is described by Eq. (3):

(3) $$Y=\beta_{A}A+\beta_{B}B+\beta_{C}C+\beta_{D}D+\beta_{\mathrm{AB}}\mathrm{AB}+\beta_{\mathrm{AC}}\mathrm{AC}+\beta_{\mathrm{AD}}\mathrm{AD}+\beta_{\mathrm{BC}}\mathrm{BC}+\beta_{\mathrm{BD}}\mathrm{BD}+\beta_{\mathrm{CD}}\mathrm{CD}+\propto_{\mathrm{EF}}\mathrm{EF}+\propto_{\mathrm{EE}}E^{2}+\propto_{\mathrm{FF}}F^{2}+\gamma_{\mathrm{AE}}\mathrm{AE}+\gamma_{\mathrm{AF}}\mathrm{AF}+\gamma_{\mathrm{BE}}\mathrm{BE}+\gamma_{\mathrm{BF}}\mathrm{BF}+\gamma_{\mathrm{CE}}\mathrm{CE}+\gamma_{\mathrm{CF}}\mathrm{CF}+\gamma_{\mathrm{DE}}\mathrm{DE}+\gamma_{\mathrm{DF}}\mathrm{DF}$$

where *Y* = response, *A* = DOPC content, *B* = DOPG content, *C* = cholesterol content, *D* = DSPE-mPEG_2000_ content, *E* = pH, *F* = lipid concentration, *α* = coefficients of process parameters, *β*_*X*_ = linear mixture coefficients, *β*_*XY*_ = binary mixture interaction coefficients, and *γ* = mixture-process interaction coefficients.

The number of runs included in the model was selected to achieve a fraction of the design space (FDS) of 0.82 at a precision of 6% and an expected standard deviation of 4%. This means that changes of at least 6% in the response can be detected with 95% confidence across 82% of the design space, assuming that the standard deviation of the response is 4%.

The resulting experimental plan consisted of two experimental blocks containing a total of 33 model points, three lack-of-fit points, and four replicates to estimate pure error. For more information, see Supporting Information. Each formulation was prepared as described above according to the experimental plan with a drug load of 5% (*n*_*drug*_*/n*_*lipids*_) and a *c(ACBI2)*_*theoretic*_ of 2.5 mM and analyzed regarding *Z*-Average, PDI, zeta potential, D/L ratio, and IE.

Data was transformed as necessary and a model reduction was performed, excluding interactions with *p*-values >0.05. Each model was evaluated based on its analysis of variance (ANOVA).

#### 2.2.8 Stress test of liposomes

The lead candidate formulations were prepared and analyzed as described above and then stored in crimped-glass vials for 1 month at 50 °C in a drying chamber. The samples were analyzed regarding their Z-Average, PDI, and zeta potential, before removing released API *via* centrifugation at 10,000 x *g* and 22 °C for 10 min. HPLC samples were prepared from the supernatant to determine the remaining drug content relative to that at t_0_.

#### 2.2.9 Cell culture

##### 2.2.9.1 Cell cultivation

A549 cells were purchased from Sigma-Aldrich (St. Louis, USA), and HeLa cells were obtained from Leibniz Institute DSMZ-German Collection of Microorganisms and Cell Cultures GmbH (Braunschweig, Germany). Both cell lines were cultured in RPMI 1640 (PAN-Biotech GmbH, Aidenbach, Germany) supplemented with 10% fetal calf serum (FCS) (Sigma-Aldrich, St. Louis, USA) at 37 °C and 5% CO_2_. The cells were passaged after reaching ∼80% confluency, and passage numbers of 3 to 20 were used for experiments. Cells were tested for mycoplasma using a Mycoplasma Detection Kit (InvivoGen Europe, Toulouse, France) after thawing and at regular intervals. All experiments involving live cells were performed under aseptic conditions.

##### 2.2.9.2 Cell viability assay

*In vitro* performance of the developed liposomal formulations was assessed through cell viability testing using a CellTiter-Glo® 2.0 Cell Viability Assay (Promega GmbH, Walldorf, Germany), which employs firefly luciferase along with luciferin to measure the ATP content within the cells. 400 cells/well within 200 μL were seeded onto white 96-well plates (Cellstar®, Greiner bio-one International GmbH, Frickenhausen, Germany). The outer wells were filled with PBS instead of cells to reduce the edge effect (Lundholt et al., 2003; Patel et al., 2005). After 24 h, the cells were treated by removing the existing medium and replacing it with treatment diluted to the desired concentrations with RPMI +10% FCS. The medium was replaced after 2 h incubation with treatment and again after three days. The assay was conducted on day 6 after treatment.

For the assay, 150 μL of the medium were removed from each well. Next, 50 μL of CellTiter-Glo ® reagent was added to the remaining 50 μL in the wells. The plate was shaken at 600 rpm and 25 °C for 10 min using a Thermomixer Comfort (Eppendorf AG, Hamburg, Germany), followed by another 10 min of incubation in the dark. Luminescence was measured using an FLx800 plate reader (BioTek Instruments, Winooski, USA) at 528/20 nm with automatic gain adjustment to the medium control wells. Each sample was tested in triplicate to sextuplicate. Viability was expressed as luminescence relative to medium control wells. Since background luminescence was below 0.1%, background subtraction was found to be negligible.

##### 2.2.9.3 BrdU cell proliferation assay

The BrdU cell proliferation assay was performed using a colorimetric BrdU Cell Proliferation ELISA assay kit purchased from Roche Diagnostics GmbH (Penzberg, Germany). This ELISA-based assay quantifies the amount of 5-bromo-2′-deoxyuridine (BrdU) incorporated into the DNA of proliferating cells.

12,000 HeLa cells or 8000 A549 cells per well were seeded in a total volume of 200 μL onto transparent 96-well plates (Cellstar®, Greiner bio-one International GmbH, Frickenhausen, Germany). The outer wells were filled with PBS to reduce the edge effect (Patel et al., 2005). Cells were treated 24 h after seeding, followed by a medium change after 2 h of incubation. BrdU was added to the wells during the medium change. After 24 h, the assay was performed according to the manufacturer's protocol. Briefly, the well plates were dried by flipping them upside down and tapping, followed by a 1 h drying step at 60 °C in a drying chamber. The cells were then fixed and denatured by adding 200 μL of FixDenat solution per well, followed by a 30 min incubation at room temperature. At the end of this step, the wells were dried by flipping and tapping. Next, anti-BrdU-peroxidase antibodies (anti-BrdU-POD) were diluted and added to the wells, according to the manufacturer's protocol. The cells were incubated with the antibodies for 90 min at room temperature. The wells were then washed three times using the washing solution (PBS) of the assay kit: the first washing step was performed with 300 μL, and the other washing steps with 200 μL per well. Between these steps, the wells were emptied by flipping and tapping. The washed wells were treated with substrate solution and incubated at room temperature for 30 min before stopping the reaction with 25 μL of 1 M sulfuric acid. The absorbance of the wells was measured at dual wavelengths of 450 and 750 nm. Each sample was analyzed in sextuplicate.

For evaluation, the absorption at 750 nm was subtracted from that at 450 nm. Two types of controls were included in each experiment: a blank for the background noise, which did not contain any cells, and a control that was not treated with BrdU to monitor nonspecific binding of anti-BrdU-POD antibodies to the cells. The averaged blank value was subtracted from the measured absorption difference of each sample, and proliferation was expressed relative to the untreated medium controls.

## 3 Results and discussion

### 3.1 HPLC – Method development and validation

An HPLC-DAD-CAD method was developed to quantify ACBI2 and other PROTACs, along with the lipids used for the DoE-based formulation development. Due to higher sensitivity, ACBI2 and other PROTACs were quantified using a diode array detector (DAD). Lipids, which do not contain chromophores, were detected with a charged aerosol detector (CAD).

**Fig. 1:**
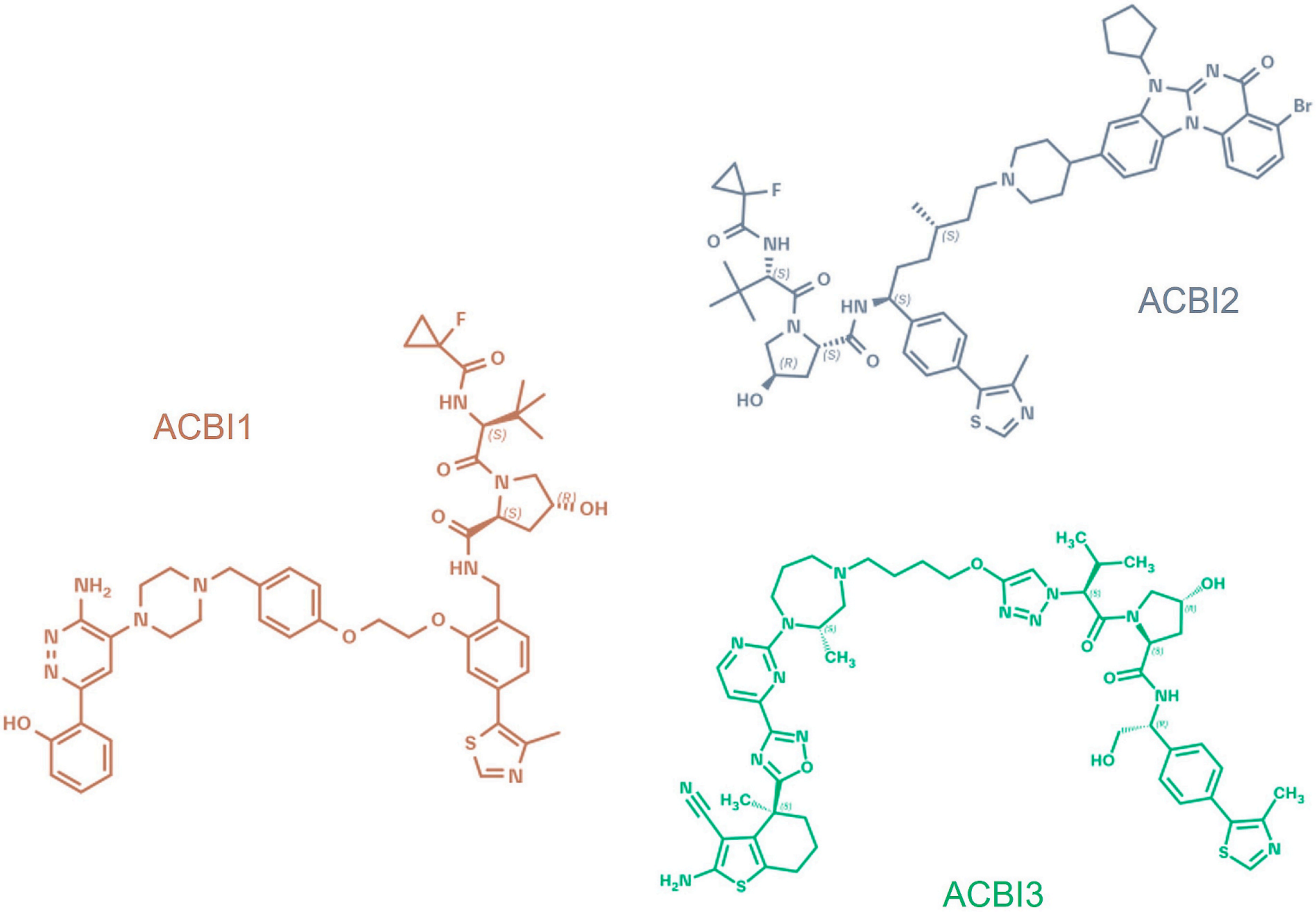
Chemical structures of the PROTACs ACBI1, ACBI2, and ACBI3. ACBI1 is an unselective PROTAC against SMARCA2 and SMARCA4. ACBI3 is a pan-KRAS degrader (Popow et al., 2024a).

The HPLC method was originally developed for ACBI1 and successfully employed for ACBI2 and ACBI3, demonstrating the wide applicability of the method for PROTACs and other chameleonic, beyond Ro5 drugs (Fig. 1).

**Fig. 2:**
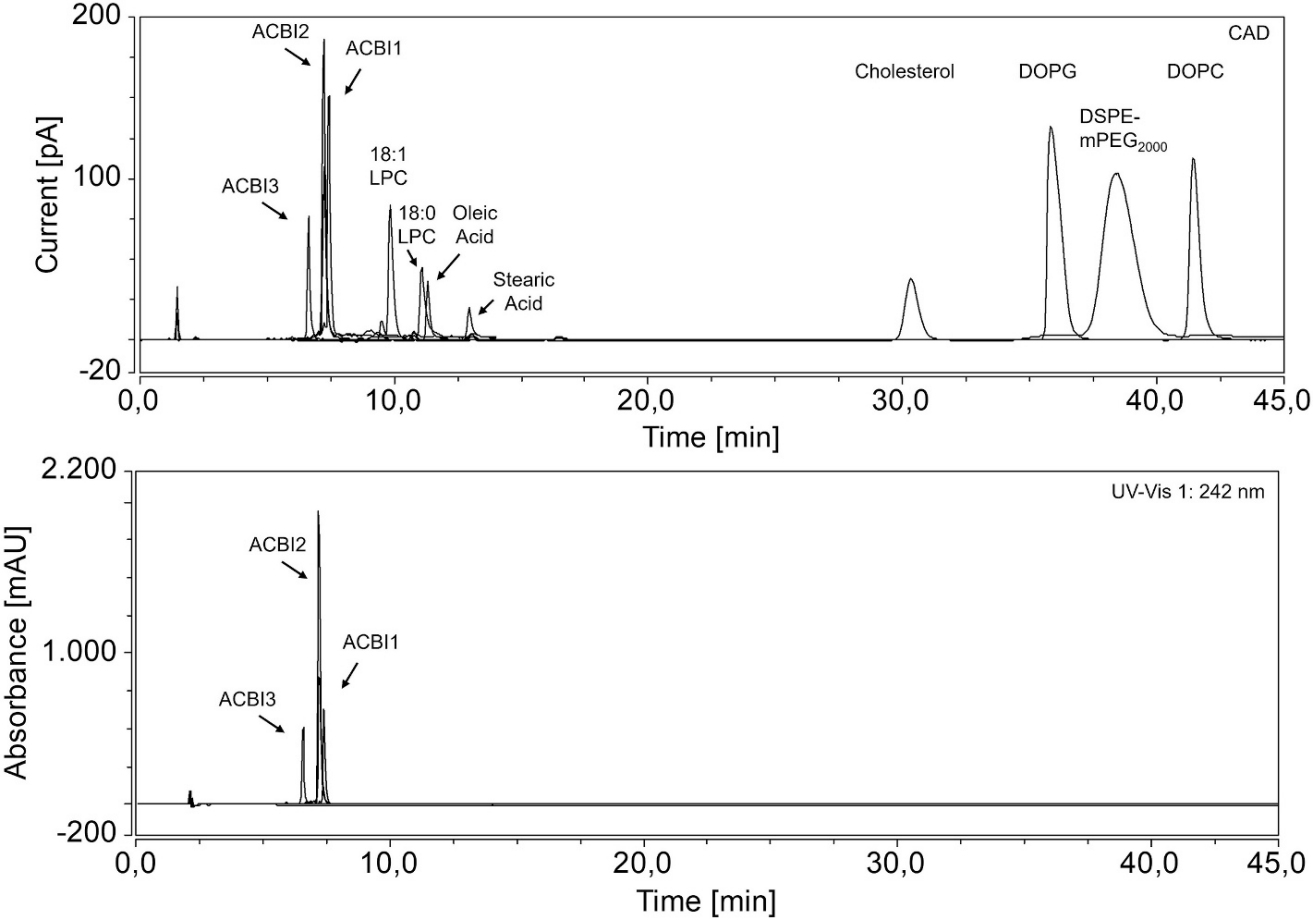
Overlaid chromatograms of all components used in this study. The chromatogram at the top shows the chromatograms derived from the CAD. At the bottom, the absorbance of the PROTACs ACBI1, ACBI2, and ACBI3 is shown, detected at 242 nm with the DAD.

A chromatogram demonstrating the retention and separation of the APIs and formulation components is shown in Fig. 2. The HPLC method was developed to separate PROTACs from liposomal components; therefore, separation of individual PROTACs was not aimed for.

The method was successfully validated in accordance with ICH guideline Q2(R2) (European Medicines Agency (EMA), 2023). It demonstrated excellent specificity, high accuracy, adequately low limits of detection and quantification and satisfactory precision. Additionally, liposomes prepared by dual centrifugation were analyzed, and a high recovery of the components was achieved. Furthermore, no process-related degradation products were detected (see Fig. A.6). For more details, see Supporting Information (Fig. A.1-A.6, Table A.1-A.6).

### 3.2 DoE-based screening for ACBI2-liposomes

#### 3.2.1 Model evaluation

The DoE was conducted according to the experimental plan (Table A.9) and the individual results are shown in Table A.10. The data was entered into the Stat-Ease 360 software, and each response was transformed as necessary. Non-significant (*p* > 0.05) terms were excluded from Eq. (3), see Section 2.2.7., and an analysis of variance (ANOVA) of each resulting model was performed. Data transformation, included interactions, and ANOVA results are presented in Table A.11. All models were significant, with a *p*-value <0.0001 and a non-significant lack of fit (*p* ≥ 0.2554). This means that all terms included in the models explain variations in the response that significantly differ from noise. A non-significant lack of fit indicates that the variation of the design points around their model-predicted values is not detectably larger than the pure error estimated from the replicates. This means that there is no statistical evidence that the model does not fit the data.

Further evaluations of the models are presented in Table 2. All models, except the PDI model, had high R^2^ values (> 0.8) and similar adjusted R^2^ values, meaning that most response variation can be explained by the model and that it does not contain unnecessary terms that artificially inflate the R^2^. In all cases, the difference between adjusted R^2^ and predicted R^2^ was <0.2, also indicating no overfitting of the model. All types of R^2^ values were lower for the PDI model, indicating that a smaller portion of the total variance from noise can be explained by the model. Nevertheless, it remains a valid model. High adequate precision (> 9.6) signifies that all models were precise enough to distinguish signal from noise. More information on the model equations, residual plots, predicted *vs.* actual plots, and a breakdown of the experimental timeline and API consumption is available in the Supporting Information (Table A.12, A.13, Fig. A.7, A.8).

**Table 2:** Fit Statistics of the obtained models. Each response is reported, including standard deviation (SD), mean, coefficient of variation (C.V.), R^2^, adjusted R^2^, predicted R^2^, and adequate precision (Adeq Precision). Data are included in transformed format.

	*Z*-Average	PDI	Zeta Potential	D/L-Ratio	IE
SD	0.0072	0.2326	162.39	0.6725	9.34
Mean	0.0859	−1.64	1072.63	4.32	63.03
C.V. %	8.37	14.21	15.14	15.58	14.82
R^2^	0.8365	0.6453	0.9326	0.7924	0.8769
Adjusted R^2^	0.7983	0.5474	0.911	0.7522	0.8482
Predicted R^2^	0.7244	0.3579	0.8646	0.6866	0.7851
Adeq Precision	15.8768	9.6954	21.334	14.3622	21.0967

#### 3.2.2 DoE models

**Fig. 3:**
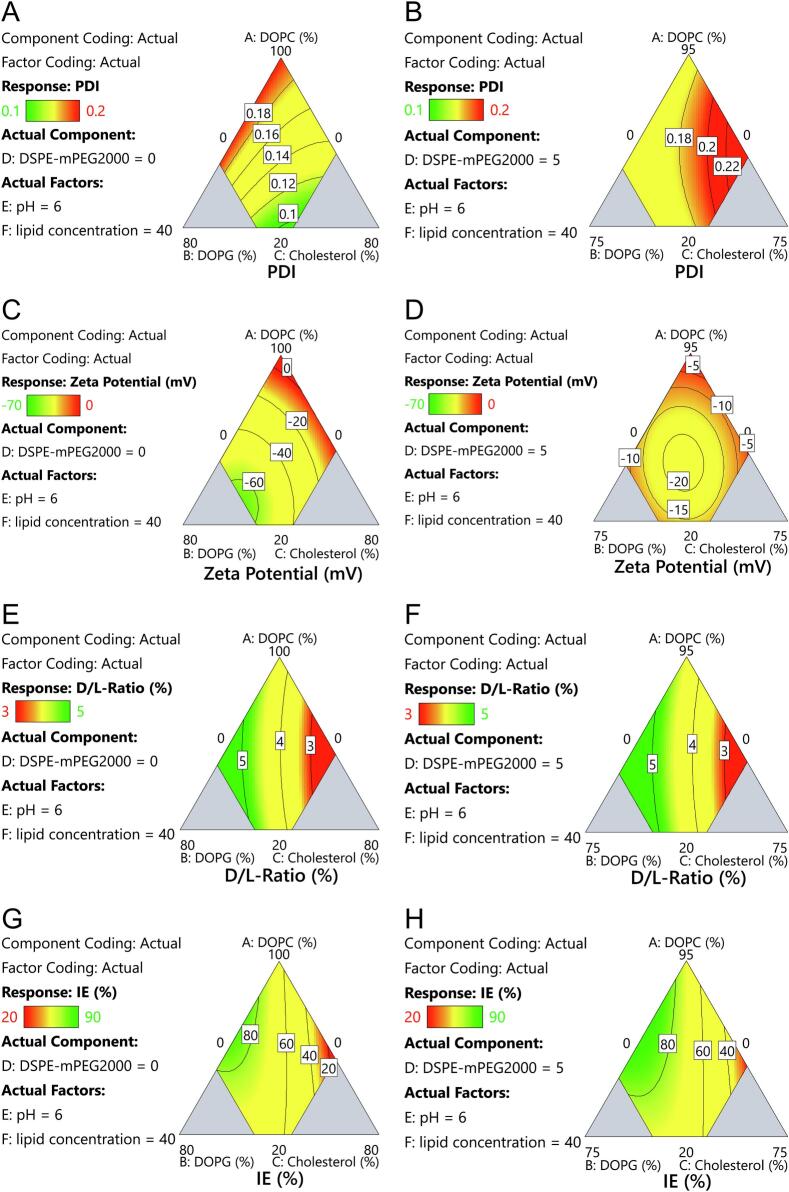
Ternary mixture diagrams of the predicted responses derived from the DoE. Each response is shown without (left side) and with 5% DSPE-mPEG_2000_ (right side). (A) and (B) show PDI, (C) and (D) show zeta potential, (E) and (F) show D/L ratio, and (F) and (G) show IE.

**Fig. 4:**
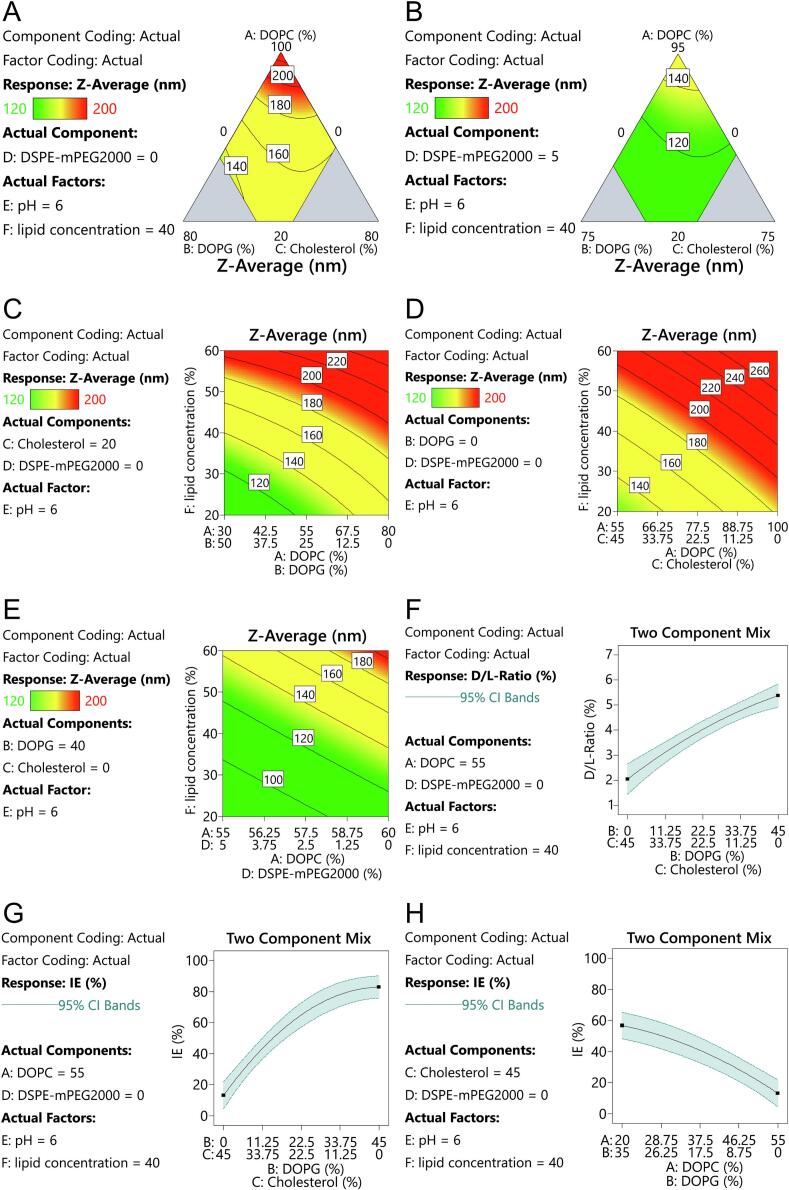
Model diagrams derived from the DoE. (A) and (B) show ternary mixture diagrams of the *Z*-Average model without (A) and with 5% (B) DSPE-mPEG_2000_. (C), (D), and (E) show further model diagrams of the Z-Average model: (C) shows the interaction between the DOPC and DOPG content and the lipid concentration, (D) shows the influence of DOPC and cholesterol content and lipid concentration, and (E) describes the interaction of the DOPC and DSPE-mPEG_2000_ content and lipid concentration. The impact of DOPG and cholesterol content on the D/L ratio (F) and IE (G) is displayed. (H) shows the influence of DOPC and DOPG content on IE.

Ternary mixture diagrams of the predicted responses for PDI, zeta potential, D/L ratio, and IE are shown in Fig. 3, further model graphs are presented in Fig. 4 and Fig. A.9.

The PDI was generally higher when the lipid mixture contained DSP*E*-mPEG_2000_. Conversely, a high cholesterol content in the absence of PEG-lipids resulted in low PDI values (Fig. 3 A,B). In PEG-free liposomes, the zeta potential was primarily influenced by the DOPG content. This was expected, as DOPG is a negatively charged phospholipid, whereas DOPC and cholesterol are neutral (Fig. 3 C). The incorporation of DSPE-mPEG_2000_ consequentially led to charge masking by the PEG chains, resulting in a moderately negative charge of the liposomes (0 to −20 mV) with less impact of the DOPG content (Fig. 3 D). Interestingly, liposomes containing high amounts of DOPG and 5% PEG-lipid were less negatively charged than formulations with moderate DOPG and cholesterol amounts. A plausible explanation for this could be the incorporation of the API, which increased with higher DOPG and lower cholesterol content. Based on computated pKa values, ACBI2 has two protonated amines at pH 6 and is therefore positively charged (pKa values of ACBI2 computed by ChemDraw 19.1 are 9.80 and 13.30). Therefore, it may partially neutralize this charge in formulations with high drug loads.

The D/L ratio and IE were almost unchanged by the DSPE-mPEG_2000_ content in the liposomes (Fig. 3 E-H). Generally, D/L ratio followed a simple trend: the more DOPG and the less cholesterol present in the mixture, the higher the D/L ratio. This interaction was almost linear, as shown in Fig. 4 F. The same trend was observed for IE, though the curve flattens at high IE values (Fig. 4 G). This suggests that more lipid was lost in this area of the design space when formulations were purified by centrifugation. As a result, the D/L ratio increases continuously in the DOPG/cholesterol interaction, but IE reaches a plateau. In summary, the capability of the liposomes to take up ACBI2 increases steadily with increasing DOPG and decreasing cholesterol content, but this is not expressed by IE.

It is widely accepted that cholesterol has a major increasing effect on packing density of lipid bilayers and that it reduces packing defects, which are important for drug-membrane interactions (Pöhnl et al., 2023; Vanni et al., 2014). As observed from our data, ACBI2 interaction with liposomes is heavily mixture dependent, so it is plausible that ACBI2 penetrates the bilayer, at least partially. When the packing density is increased by cholesterol, there is less space for ACBI2 to fit. The reduction in packing defects caused by cholesterol can further decrease drug-phospholipid interaction opportunities and drug incorporation.

Formulations with high cholesterol content also showed a strong interaction between DOPC and DOPG: the more DOPG (at the expense of DOPC), the higher the IE (Fig. 4 H). Interestingly, this interaction was not observed in the absence of cholesterol. A high proportion of DOPG was more critical for achieving a high IE when cholesterol was present, likely due to the resulting increase in membrane packing density.

It further appeared that formulations with a high drug content had a high PDI. This interaction was checked for using Stat-Ease 360 and was found to not be significant.

*Z*-Average was reduced with increasing amounts of DOPG, cholesterol, and DSPE-mPEG_2000_ (Fig. 4 A-E). The lipid concentration during the first step of dual centrifugation had a very strong, albeit unsurprising, effect on the Z-Average (Fig. 4 C-E). This process parameter in dual centrifugation determines the lamellarity of the resulting liposomes (Koehler et al., 2023a). Consequently, higher lipid concentrations result in larger liposomes containing more lamellae. The DoE model graphs show that liposomes produced at 60% lipid concentration to obtain multilamellarity could still be considered small, indicating the successful preparation of ACBI2-SMVs.

The pH played had significant effects in all models, except the *Z*-Average (Table A.11). However, its interactions were not as pronounced as others. Higher pH values tended to result in liposomes with lower PDI, zeta potential, and IE (Fig. A.9 A-C). This is plausible because at higher pH, more lipids may be deprotonated, and because IE is also slightly lower, less ACBI2 is available to neutralize the negative charge from the phospholipids. Drug incorporation might be lower because less ACBI2 might be protonated at higher pH levels; therefore, less electrostatic interaction can occur when a lower proportion of the compound is in a charged state.

Lipid concentration did not have a strong effect on the resulting liposomes, except for their size, as discussed above. Only drug incorporation was slightly higher in unilamellar liposomes produced at low lipid concentrations (Fig. A.9 D).

This was surprising, as we expected the opposite: SMVs contain several lamellae with smaller radii and, therefore, with a stronger curvature. Strong curvature causes packing defects which allow more interaction between the lipid bilayer and the API (Vanni et al., 2014; Zhang et al., 2019). This effect is more pronounced in the absence of cholesterol, as it reduces the number of packing defects (Pöhnl et al., 2023; Zhang et al., 2019). A possible explanation for the opposite trend observed here could be the accessibility of the bilayers for ACBI2. Although dual centrifugation is a very thorough homogenization process, it is possible that less ACBI2 gets exposed to the innermost lamellae of the SMVs (Koehler et al., 2023b). A more likely explanation could be that SMVs are larger than their unilamellar counterparts (Fig. 4 C-E). As a result, the outer layers of the SMVs, which are the largest in terms of the area of the bilayer, have less curvature than the corresponding SUVs, which in turn could decrease drug incorporation.

Considering all the information, the best formulations, defined as small, a PDI below 0.2, and high drug loading, were obtained using high amounts of DOPG, low to no cholesterol, 5% DSPE-mPEG_2000_, and low lipid concentrations. The pH was maintained at 6 to balance high drug loading and high phospholipid stability.

#### 3.2.3 Model confirmation

All models were successfully confirmed at five separate locations, and each confirmation point was controlled in triplicates.

Four locations were carefully selected guided by the DoE software to maximize IE and D/L ratio while maintaining a *Z*-Average of 100 nm and a PDI < 0.2. One formulation was selected to be cholesterol-free (formulation 1: DOPC/DOPG/DSPE-mPEG_2000_ 47.5/47.5/5) and two more formulations were chosen with low, increasing amounts of cholesterol (formulation 2: DOPC/DOPG/cholesterol/DSPE-mPEG_2000_ 50.6/42/2.4/5 and formulation 3: DOPC/DOPG/cholesterol/DSPE-mPEG_2000_ 37.7/50/7.3/5). One formulation was designated to be free of DSPE-mPEG_2000_, as accelerated blood clearance effects and adverse reactions to PEG-lipids are well known (formulation 4: DOPC/DOPG/cholesterol 46.9/41.3/11.8) (Chanan-Khan et al., 2003; Dams et al., 2000; Ishida et al., 2006, Ishida et al., 2005; Szebeni, 2005; Szebeni et al., 2002). The last formulation (formulation 5: DOPC/cholesterol/DSPE-mPEG_2000_ 61.5/37/1.5) was selected as a control formulation to have a similar size, but a larger PDI > 0.2 and a low IE of <50% to confirm a different location of the design space. All formulations are shown in Table 3, including the mean derived from an experimental triplicate and the 95% confidence limits. All responses of all formulations remained within the confidence interval, which means that all models were successfully confirmed.

**Table 3:** Locations and results of the confirmation runs. Data are shown as the actual mean (*n* = 3) in comparison to the 95% prediction intervals at five different locations. All results were within the 95% prediction interval.

Formulation location #	DOPC / DOPG / Cholesterol / DSPE-mPEG_2000_	pH	c(lipid)	Analysis	95% prediction interval low	Data Mean	95% prediction interval high
1	47.5 / 47.5 / 0/ 5	6	20	*Z*-Average	65.1	95.9	96.0
				PDI	0.140	0.195	0.277
				Zeta Potential	−12.1	−10.1	5.6
				D/L-Ratio	5.13	6.01	7.14
				c(ACBI2)	1.9	2.3	2.5
				IE	74.2	90.7	100.6
2	50.6 / 42 / 2.4 / 5	6	40	Z-Average	68.8	99.5	100.5
				PDI	0.143	0.183	0.273
				Zeta Potential	−16.7	−12.3	1.4
				D/L-Ratio	5.0	5.5	6.9
				c(ACBI2)	1.9	2.3	2.6
				IE	76.2	94.7	102.5
3	37.7 / 50 / 7.3 / 5	6	40	Z-Average	82.6	90.5	122.1
				PDI	0.113	0.196	0.228
				Zeta Potential	−20.5	−12.9	−2.4
				D/L-Ratio	4.58	6.01	6.40
				c(ACBI2)	1.7	2.3	2.3
				IE	67.0	91.2	92.7
4	46.9 / 41.3 / 11.8 / 0	6	20	Z-Average	87.8	89.5	132.7
				PDI	0.138	0.146	0.254
				Zeta Potential	−58.1	−46.4	−33.0
				D/L-Ratio	4.12	5.52	5.87
				c(ACBI2)	1.7	2.3	2.3
				IE	67.6	90.7	93.2
5	61.5 / 0 / 37 / 1.5	6	20	*Z*-Average	97.0	111.9	155.0
				PDI	0.141	0.220	0.271
				Zeta Potential	−12.8	−0.1	4.8
				D/L-Ratio	1.74	2.49	3.58
				c(ACBI2)	0.4	1.0	1.0
				IE	15.5	41.3	41.8

**Fig. 5:**
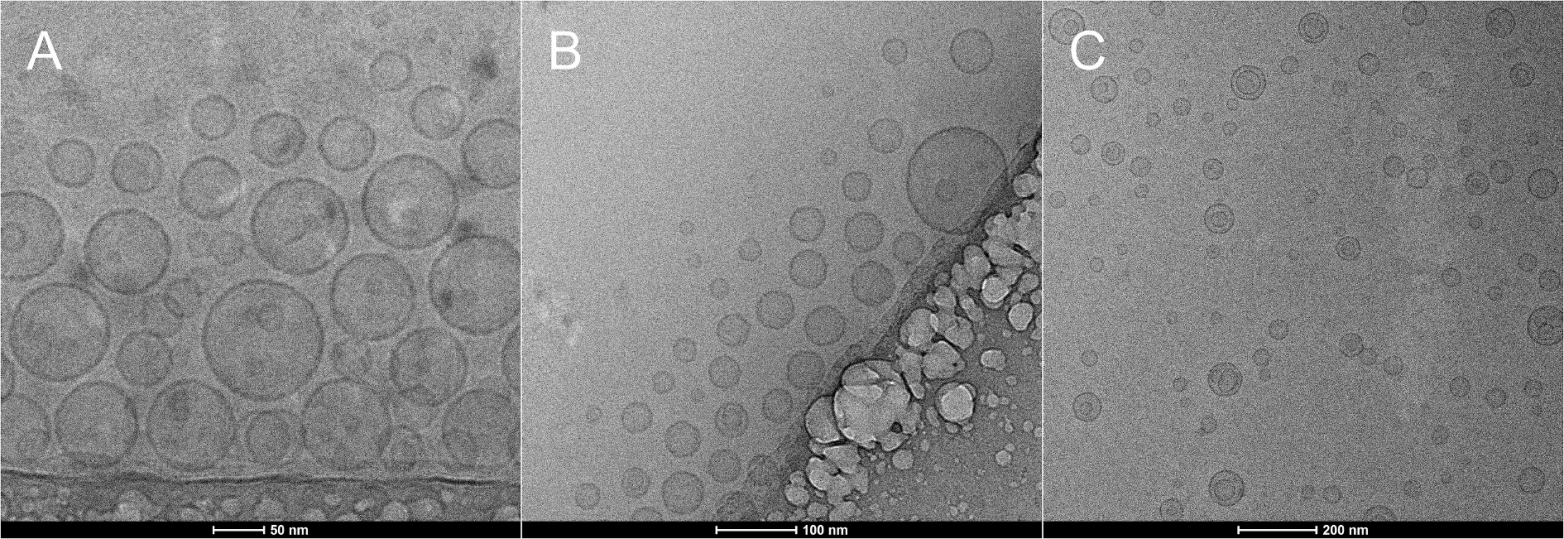
CryoTEM images of confirmation formulations derived from the DoE. (A) shows formulation 1 (DOPC/DOPG/DSPE-mPEG_2000_ 47.5/47.5/5); (B) shows formulation 2 (DOPC/DOPG/cholesterol/DSPE-mPEG_2000_ 50.6/42/2.4/5); and (C) shows formulation 4 (DOPC/DOPG/cholesterol 46.9/41.3/11.8). Scale bars: (A): 50 nm, (B) 100 nm, and (C): 200 nm.

CryoTEM images of selected confirmation formulations produced in 20 mM histidine 270 mM glucose buffer, were prepared to assess the morphology of ACBI2-loaded liposomes (Fig. 5). These images show typical liposomes with a size slightly below 100 nm, which is in good agreement with the Z-Average measured in this buffer (Fig. A.10). Unilamellar liposomes were obtained with formulation 1 (DOPC/DOPG/DSPE-mPEG_2000_ 47.5/47.5/5), as expected for manufacturing at a 20% lipid concentration (Fig. 5 A). The same lipid concentration yielded more multilamellar liposomes in the case of the non-PEGylated formulation (DOPC/DOPG/cholesterol 46.9/41.3/11.8, Fig. 5 C). PEG-lipids distance the bilayers through their high water binding capacity; therefore, PEGylated liposomes are more unilamellar than liposomes without PEGylation (Koehler et al., 2023b, Koehler et al., 2023a, Koehler et al., 2021). PEGylated liposomes prepared at a 40% lipid concentration (DOPC/DOPG/cholesterol/DSPE-mPEG_2000_ 50.6/42/2.4/5, Fig. 5 B) were mostly unilamellar but also contained some multilamellar liposomes, as expected.

**Fig. 6:**
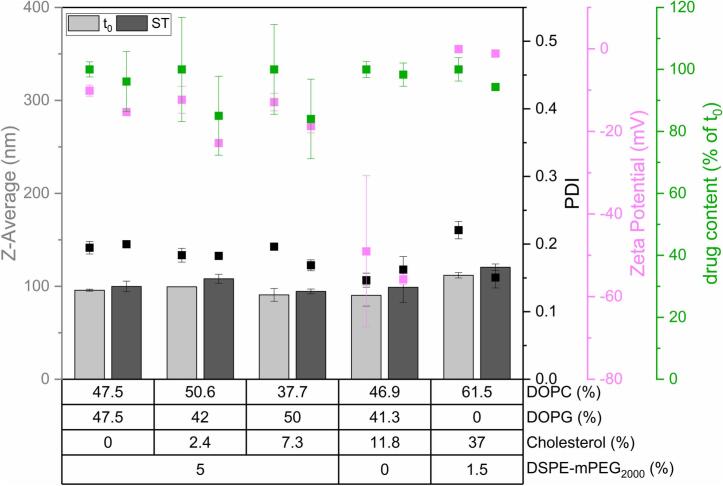
Stability data of confirmation formulations subjected to a stress test at 50 °C for 1 month (t_0_ = characteristics upon production, ST = characteristics after stress test). Data are shown as mean ± SD, *n* = 3.

The confirmation formulations were further subjected to a stress test of 1-month storage at 50 °C to obtain initial stability estimates. The results are displayed in Fig. 6 and encouraged further advancement of these formulations.

### 3.3 Impact of lipid head groups and saturation on ACBI2-liposomes

In this section, the impact of lipid exchange on ACBI2-liposomes was investigated. For this purpose, the formulations derived from the DoE were examined, and DOPC was exchanged with DOPE. In a second experiment, both DOPC and DOPG were replaced by DSPC and DSPG, respectively.

DOPE is a zwitterionic phospholipid like DOPC, but it possesses a different molecule geometry. DOPC is a cylindrical phospholipid that forms lamellar structures, whereas DOPE has a small polar headgroup and consequently an inverted cone shape, resulting in the formation of inverted hexagonal structures (Campbell et al., 2001; Du et al., 2014; Israelachvili et al., 1976; Kumar, 1991). As a result, the introduction of DOPE into a lipid mixture can favor negative curvature and is frequently used in nucleic acid delivery to promote endosomal escape (Cullis and Felgner, 2024; Du et al., 2014). We were particularly interested in investigating the impact of packing defects introduced by the higher curvature of DOPE on the incorporation of ACBI2 into the membrane.

**Fig. 7:**
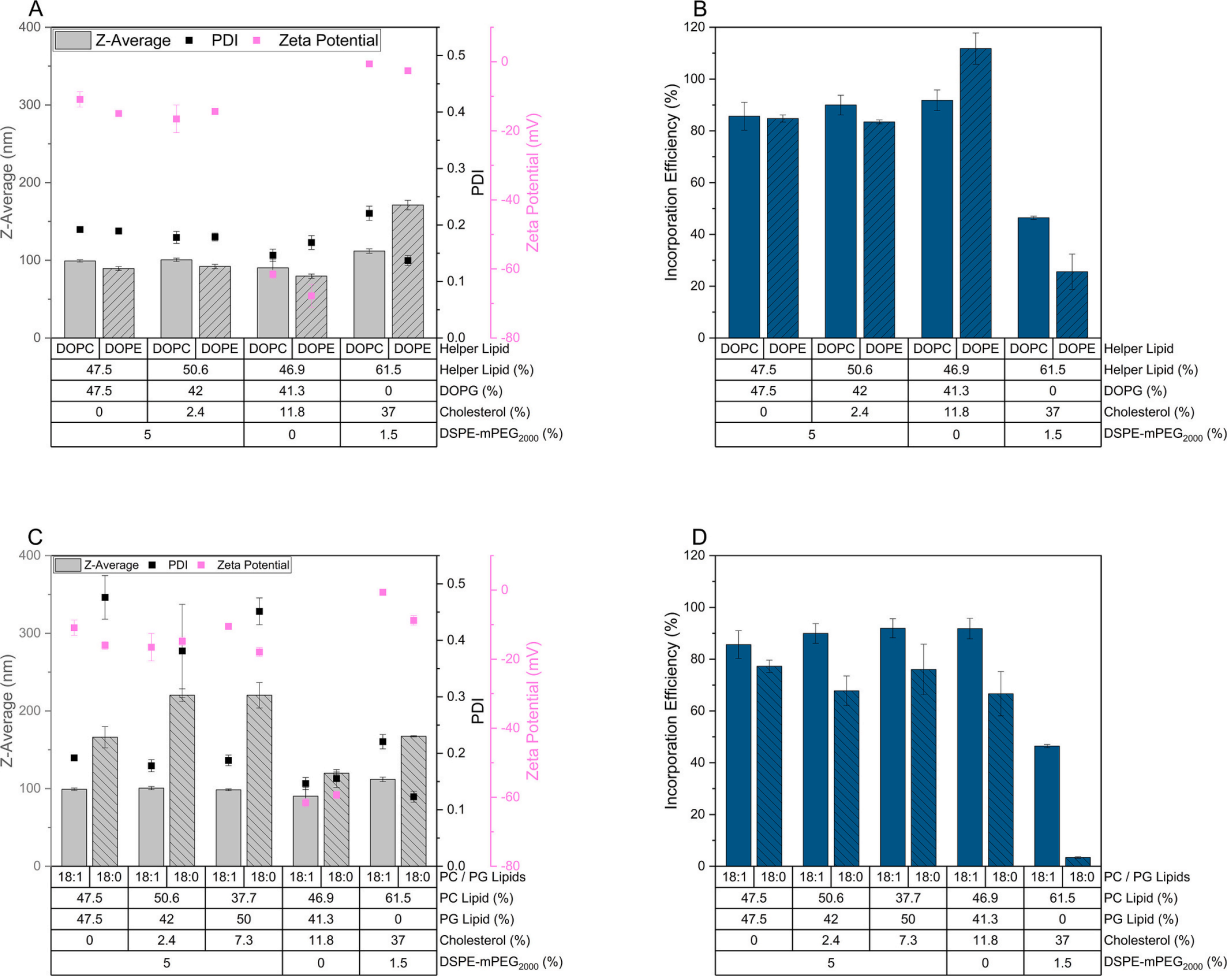
Phospholipid modifications. (A) and (B): DOPC *vs.* DOPE liposomes. (A) Z-Average, PDI, and zeta potential and (B) incorporation efficiency of the DoE-derived formulations prepared in histidine-glucose buffer compared to the same formulations with DOPE instead of DOPC. (C) and (D): Investigation of the influence of lipid saturation on ACBI2-liposomes. The DoE-derived formulations were compared to identical compositions in which the unsaturated (18:1) PC and PG lipids were replaced with their saturated (18:0) counterparts. (C) shows Z-Average, PDI, and zeta potential. (D) shows incorporation efficiency of these liposomes prepared in histidine glucose buffer. Data are shown as mean ± SD, n = 3.

The characteristics of DOPE liposomes compared to DOPC liposomes are shown in Fig. 7 A,B. Z-Average, PDI, and zeta potential were similar in the first three formulations (Fig. 7A). The control formulation with DOPE was larger and had a lower PDI than the associated DOPC formulation. Drug incorporation did not follow a uniform trend (Fig. 7 B). However, DOPE addition did not cause a coherent increase in IE.

In a subsequent experiment, DOPC and DOPG were replaced with their saturated counterparts, DSPC and DSPG, respectively, to investigate the impact of lipid saturation and membrane rigidity on drug incorporation. Here, the trend was more discernible: liposomes produced with saturated lipids exhibited an overall lower IE than their DOPC/DOPG based counterparts (Fig. 7 D). Liposomes prepared with saturated lipids were also less favorable in terms of their size characteristics; they were larger and had a higher PDI in most cases (Fig. 7 C). The decrease in IE in saturated, rigid membranes is consistent with the IE-lowering effect of cholesterol, which is attributed to its increasing influence on bilayer packing density.

### 3.4 ACBI2 incorporation

We further investigated the mode of ACBI2 incorporation into the liposomes.

**Fig. 8:**
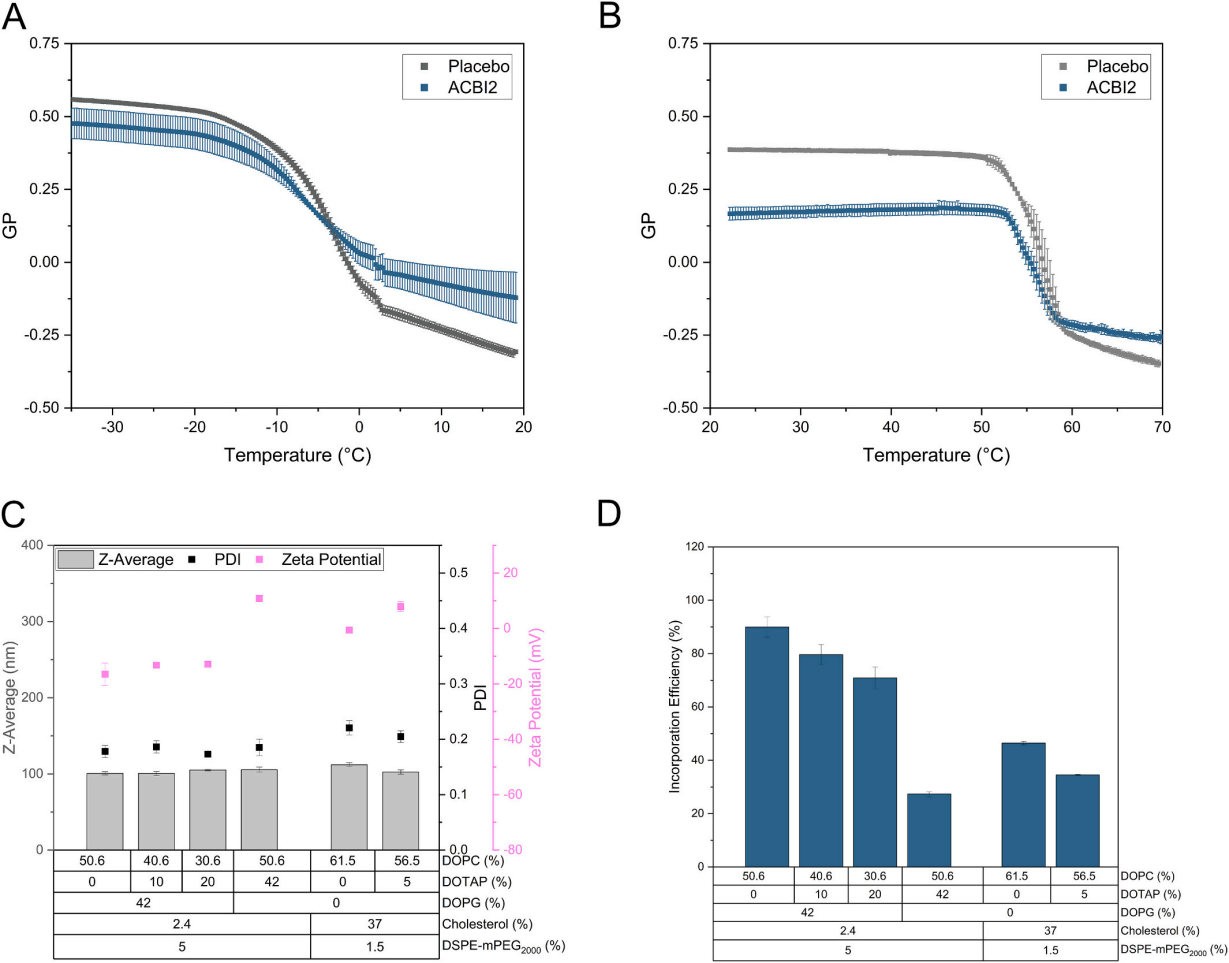
(A) and (B): LISO data. GP against the temperature of formulation 2 (PC/PG/cholesterol/DSPE-mPEG_2000_ 50.6/42/2.4/5) using (A) unsaturated (18:1) and (B) saturated (18:0) PC and PG lipids. Both formulations were analyzed without ACBI2 (“Placebo”) and with 5% (n/n) ACBI2 (“ACBI2”). (C) and (D): Impact of cationic DOTAP on ACBI2-liposomes. (C) shows Z-Average, PDI, and zeta potential and (D) incorporation efficiency of formulation 2 and 5 derived from the DoE (DOPC/DOPG/cholesterol/DSPE-mPEG_2000_ 50.6/42/2.4/5 and 61.5/0/37/1.5). Data are shown as mean ± SD, n = 3.

The DoE results strongly suggest at least partial integration of ACBI2 into the lipid bilayer. Consequently, we aimed to investigate how this interaction alters the lipid bilayer and to confirm the interaction between ACBI2 and the lipid bilayer. This objective was pursued with LISO measurements of formulation 2 (PC/PG/cholesterol/DSPE-mPEG_2000_ 50.6/42/2.4/5) using unsaturated (18:1) and saturated (18:0) PC and PG lipids. Each formulation was analyzed with empty liposomes and with ACBI2-liposomes with a drug load of 5% (*n/n*). The resulting plots showing *GP* values against temperature are shown in Fig. 8 (A: 18:1 lipids, B: 18:0 lipids). High *GP* values signify highly ordered bilayers, whereas low *GP* values are measured from bilayers with low ordering. Phase transition occurs at the turning point of the curves.

The same trend was observed for both formulations: the lipid order of ACBI2-liposomes was lower in the gel phase and higher in the liquid crystalline phase than that of empty liposomes. There was no impact on the phase transition temperature itself. This suggests that ACBI2 at least partially inserts into the lipid bilayer, thereby exerting an effect similar to that of cholesterol on lipid bilayers (Färber and Westerhausen, 2022). The DoE data further emphasized the importance of DOPG in the lipid mixture for achieving high drug incorporation. This is attributed to a suspected electrostatic interaction between the protonated nitrogen of ACBI2 and the anionic phosphate group of DOPG, as previously described for other drugs (amphotericin B, berberine, and dipyridamole) and PG lipids (Adler-Moore and Proffitt, 2002; Dhakal et al., 2025; Nassar et al., 1998).

In a control experiment, formulation 2 from the DoE (DOPC/DOPG/cholesterol/DSPE-mPEG_2000_ 50.6/42/2.4/5) was prepared, with 10% and 20% of DOPC being replaced with DOTAP. As DOTAP is a cationic lipid with a molecular shape similar to that of DOPC and DOPG, this substitution was primarily intended to modulate the surface charge. Similar charge-tuning of liposomes has been reported before (Wang et al., 2023). *Z*-Average, PDI, and zeta potential remained comparable, and no particle aggregation was observed (Fig. 8 C); however, the IE decreased despite the similar zeta potential and high DOPG content (Fig. 8 D). To further enhance this effect, all DOPG was replaced with DOTAP. This resulted in liposomes with similar size characteristics but a positive zeta potential and a dramatically reduced IE. Moreover, the DOPG-free control formulation derived from the DoE (DOPC/DOPG/cholesterol/DSPE-mPEG_2000_ 61.5/0/37/1.5) was selected, and 5% of DOPC were replaced with DOTAP. This minor modification strongly reduced the IE, corresponding to a shift of the zeta potential to positive values, while keeping the Z-Average and PDI nearly unchanged. (Fig. 8). In summary, this experiment demonstrated that adding even small amounts of DOTAP to neutral liposomes reduced ACBI2 incorporation. Furthermore, a similar decrease in loading occurred even in the presence of 42% DOPG, provided that sufficient DOTAP was added to the mixture to oppose the anionic charge. As the zeta potential was the only parameter varied, these results strongly suggest that electrostatic interactions are the primary driver of high ACBI2 incorporation in the presence of DOPG.

### 3.5 *In vitro* evaluation of ACBI2-liposomes

**Fig. 9:**
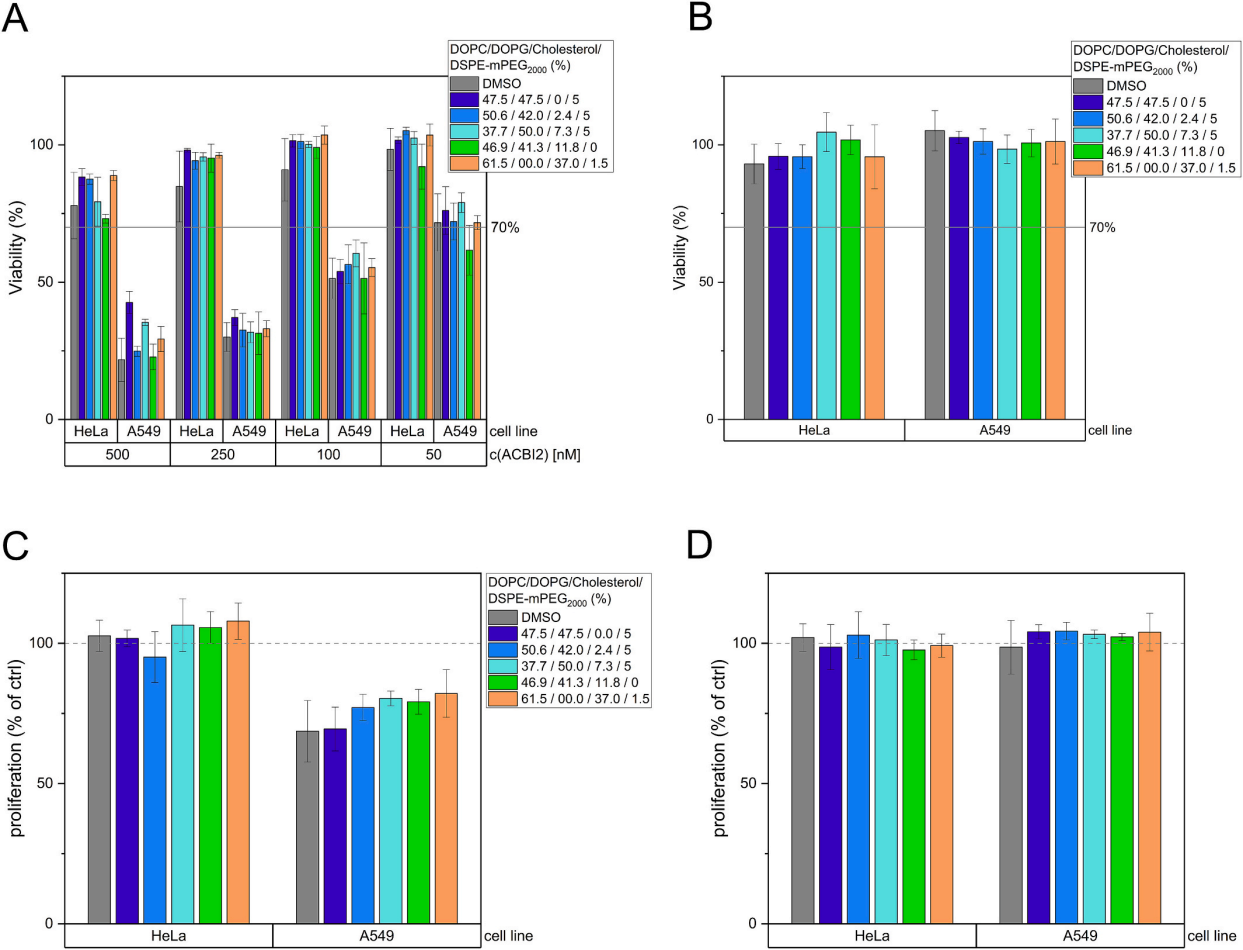
(A) and (B): Results of the CellTiter-Glo® 2.0 viability assay. 400 cells/well were seeded onto 96-well plates and treated for 2 h after 24 h of incubation. The assay was performed six days after treatment. (A) Concentration-dependent reduction of cell viability in A549 cells caused by ACBI2-liposomes and no decrease in viability in HeLa cells at these concentrations. This effect was independent of the formulation and comparable to that of the DMSO control. (B) Viability of HeLa and A549 cells after treatment with empty vehicles. No reduction in viability was observed. (C) and (D): Impact of ACBI2 on cell proliferation. (C) The results of the BrdU assay from A549 and HeLa cells treated with 250 nM ACBI2 in the DoE-derived liposomes *vs.* DMSO are shown. (D) BrdU assay results of the same cell lines treated with equal amounts of empty liposomes and DMSO. 8000–12,000 cells/well were seeded onto 96-well plates and treated for 2 h after 24 h. BrdU was added, and the assay was performed 24 h later. Data are shown as mean ± SD, n = 3.

Finally, the biological activity of the ACBI2-liposomes was evaluated using the CellTiter-Glo® 2.0 cell viability assay. This assay quantifies the amount of ATP in the well, which is directly proportional to the number of metabolically active cells. ACBI2 is an anticancer drug that selectively degrades SMARCA2 over SMARCA4, thereby exerting selective toxicity toward SMARCA4-deficient cancer cells (Diao et al., 2026; Harling and Tinworth, 2023; Hoffman et al., 2014; Kofink et al., 2022; Wilson et al., 2014). Hence, SMARCA4-deficient A549 cells were used to check for activity and SMARCA4-proficient HeLa cells to control for selectivity (Kofink et al., 2022; Vangamudi et al., 2015). The viability results of ACBI2-loaded liposomes following a 6-day incubation period after treatment are shown in Fig. 9 A. The viability of A549 cells decreased in a concentration-dependent manner, irrespective of the formulation, and was comparable to that of the free API (in DMSO). This suggests that the biological activity of ACBI2 was fully maintained upon incorporation into liposomes. The viability of HeLa cells remained unaffected across this concentration range, demonstrating the selectivity of the treatment. Empty liposomes and DMSO controls at the highest concentration showed no effect on cell viability, confirming the biocompatibility of the carrier system (Fig. 9 B).

Furthermore, we investigated the impact of lipid bilayer rigidity on biological activity. Therefore, we evaluated the viability of A549 cells after treatment with 250 nM ACBI2 in formulation 2 (DOPC/DOPG/cholesterol/DSPE-mPEG_2000_ 50.6/42/2.4/5) compared to the same formulation with saturated (18:0) lipids. The results indicate that membrane rigidity did not influence the therapeutic effect (Table A.14).

SMARCA2 degradation in SMARCA4-deficient cell lines results in a synthetic lethal effect, which disables the BAF complex (Hoffman et al., 2014; Wilson et al., 2014). As this complex has a multitude of functions in cell cycle and proliferation, its effective knockdown leads to cell cycle arrest and induction of senescence (Hoffman et al., 2014). Selective SMARCA2-degradation by PROTACs induced tumor stasis, indicating an antiproliferative effect of ACBI2 rather than a cytotoxic effect (Cantley et al., 2022; Kofink et al., 2022). This mode of action was controlled using a BrdU proliferation assay following treatment with 250 nM ACBI2 in the respective formulations. The assay was performed one day after treatment, during which the cells were incubated with BrdU. The results indicated an antiproliferative effect of ACBI2 in A549 cells (Fig. 9 C). In contrast, ACBI2 formulations showed no effect on cell proliferation in HeLa cells. Furthermore, empty formulations had no impact on either cell line (Fig. 9 C,D).

## 4 Conclusion

This study demonstrates the successful implementation of a combined mixture-process design of experiments using dual centrifugation as a fast and efficient manufacturing platform for the screening of liposomal formulations containing poorly soluble drugs such as PROTACs. A full screening was achievable within two weeks using less than 100 mg API, including confirmation runs, and achieving relevant drug loads as indicated by D/L ratios of 5.5–6.0% (*n/n*). This approach was supported by a validated HPLC-DAD-CAD method for the simultaneous quantification of all liposomal components and the API. Furthermore, the HPLC method was successfully implemented for three different PROTACs, indicating its broad applicability to a variety of poorly soluble drugs.

The screening study was performed for the PROTAC ACBI2, yielding valuable insights into its incorporation into liposomes and highlighting the necessity of a systematic formulation approach. ACBI2 incorporation followed a near-linear trend, increasing with increasing DOPG and decreasing cholesterol content in the lipid mixture. This trend was likely driven by two complementary mechanisms. On the one hand, the PROTAC electrostatically interacts with anionic DOPG *via* its protonated nitrogen atoms. This hypothesis was further strengthened by the addition of cationic DOTAP to the lipid compositions: increasing DOTAP content strongly reduced ACBI2 incorporation, even in the presence of DOPG, indicating that electrostatic interactions contribute substantially to membrane association. On the other hand, ACBI2 was demonstrated to insert at least partially into the lipid bilayer and exert a cholesterol-like effect on lipid ordering. Thus, higher lipid packing density and decreased packing defects were found to be counter-effective for ACBI2 incorporation. Consequently, more rigid membranes also counteracted drug incorporation, as demonstrated by the use of saturated (18:0) lipids in the formulations. Saturated lipids showed no benefit over unsaturated (18:1) lipids in terms of size characteristics, drug loading, and *in vitro* performance. Replacing DOPC with DOPE, a lipid with a smaller polar headgroup, intended to introduce more packing defects and increase the interaction space for ACBI2, did not yield consistent results regarding drug incorporation.

Four formulations with favorable size characteristics and high drug loading, along with one control formulation, were selected from the DoE data for further *in vitro* evaluation. All formulations performed comparably to the free API (in DMSO). No biological activity was lost by incorporating ACIB2 into a nanocarrier, and an anti-proliferative effect was demonstrated. However, further investigation of these formulations is required to draw conclusions about potential pharmacokinetic advantages, as well as the storage stability of these particles over an extended time period. This was not studied here, as it was beyond the scope of the delivery platform presented in this article.

Ultimately, this work establishes the combined DoE–dual centrifugation approach as a highly resource-efficient and predictable engineering workflow. By drastically compressing development timelines and minimizing material consumption, this integrated platform provides a powerful methodological standard for accelerating the preclinical formulation and analytical screening of challenging, poorly soluble modalities.

## CRediT authorship contribution statement

**Miriam Jaki:** Writing – original draft, Visualization, Methodology, Investigation, Conceptualization. **Monika Köll-Weber:** Writing – review & editing, Methodology, Investigation. **Jan Lembeck:** Writing – original draft, Investigation. **Maximilian Wittmann:** Writing – review & editing, Supervision, Conceptualization. **Regine Süss:** Writing – review & editing, Supervision, Resources, Project administration, Conceptualization.

## Declaration of competing interest

The authors declare the following financial interests/personal relationships which may be considered as potential competing interests:

Maximilian Wittmann reports a relationship with Boehringer Ingelheim Pharma GmbH & Co.KG that includes: employment. If there are other authors, they declare that they have no known competing financial interests or personal relationships that could have appeared to influence the work reported in this paper.

## Data Availability

The data that support the findings of this study are available from the corresponding author upon reasonable request.
