# Supplementary material for: A DoE-Dual centrifugation-driven screening platform for liposomal incorporation of poorly soluble APIs: Application to the PROTAC ACBI2
Source: Int J Pharm X. 2026 Aug 1;12:100627. doi: 10.1016/j.ijpx.2026.100627 (PMC13470561; doi:10.1016/j.ijpx.2026.100627)
Supplement: Supplementary file 1 [file mmc1.docx]

**Supporting Information: A DoE-Dual Centrifugation-Driven Screening Platform for Liposomal Incorporation of Poorly Soluble APIs: Application to the PROTAC ACBI2**

Table A.1: Summary of key characteristics for the prediction of bioavailability of poorly soluble drugs including the PROTAC ACBI2. Ro5 drugs are characterized by a molecular weight (MW) < 500 Da, a (calculated) partition coefficient (LogP/CLogP) < 5, < 10 hydrogen-bond acceptors (HBA), and < 5 hydrogen-bond donors (Lipinski et al., 2001). An extension of these rules by Veber et al. (2002) further proposes a total polar surface area (tPSA) ≤ 140 Ǻ^2^ and ≤ 10 rotatable bonds.

|  | ACBI2 | Ibuprofen | Everolimus | Paclitaxel | Amphotericin B |
| --- | --- | --- | --- | --- | --- |
| MW (Da) | 1064 | 206.29 | 958.24 | 853.92 | 923.49 |
| solubility in water | < 1 µg/mL (pH 7) (Kofink et al., 2022) | 0.06 mg/mL (Shaw et al., 2005) | < 0.1 mg/mL (FDA, 2012) | 1.16 µg/mL (Yang et al., 2020) | < 1 µg/mL (Adediran et al., 2009) |
| tPSAa (Ǻ^2^) | 150.25 | 37.3 | 204.66 | 221.29 | 319.61 |
| CLogP^a^ | 10.343 | 3.679 | 7.103 | 4.731 | -3.654 |
| rotatable bonds^b^ | 16 | 4 | 9 | 14 | 3 |
| HBA^b^ | 9 | 2 | 14 | 14 | 18 |
| HBD^b^ | 3 | 1 | 3 | 4 | 12 |

a = computed by ChemDraw 19.1, b = computed by Cactvs 3.4.8.24

# 1. HPLC Method Validation

The HPLC method was originally developed for ACBI1-liposomes and was later transferred to ACBI2- and ACBI3-liposomes. Therefore, the method was validated in accordance with ICH Guideline Q2(R2) for all lipid components, plus ACBI2 or ACBI3, and the results are shown below (European Medicines Agency (EMA), 2023).

1.1. Method Development

To achieve the retention of PROTACs on a reversed-phase column, it was necessary to start with a high water content in the eluents. When using isocratic conditions and a water content > 20%, thus achieving retention, the PROTAC eluted in more than one peak. This phenomenon might be explained by the chameleonic behavior of PROTACs: owing to their function as heterobifunctional linker molecules, PROTACs are comparably large (~ 1 kDa), flexible, and thus able to adapt their conformation to their surroundings (Atilaw et al., 2021; Békés et al., 2022; Chen et al., 2022; Ermondi et al., 2020; Rossi Sebastiano et al., 2022; Whitty et al., 2016). In apolar surroundings, chameleonic molecules can shield polar groups *via* intramolecular hydrogen bonds and π–π interactions. In polar environments, these molecules can stretch out and maximize polarity (Atilaw et al., 2021; Rossi Sebastiano et al., 2018). Therefore, the same PROTAC can exist in several conformations. This has already been demonstrated for another VHL-based PROTAC, which appeared in up to five different conformations in the same solvent (Atilaw et al., 2021). These conformations can have different distribution coefficients on the column and, therefore, elute at different times.

The addition of salts to the mobile phase (formic acid, trifluoroacetic acid, ammonium acetate) combined with the application of a steep gradient from polar to apolar eluents mitigated this problem. The presence of polar additives in the mobile phase resulted in the earlier elution of the PROTACs. This is plausible because PROTACs adopt more polar conformations in eluents with higher polarity, causing them to elute more rapidly on a reversed-phase column.

The separation of cholesterol, DOPG, DSPE-mPEG_2000_, and DOPC was achieved using a high methanol content in the mobile phase. The peak of DSPE-mPEG_2000_ was wider than that of the other peaks owing to the molecular weight distribution of the PEG chain. DSPE-mPEG_2000_ required a larger counter-ion than formic acid and its peak shape was best at highest methanol proportions, which is in line with previous work (Bender et al., 2024; Schönherr et al., 2009; Shibata et al., 2013). The addition of 4 mM ammonium acetate was vital for PG-lipid detection, possibly because it required a positively charged counter-ion for ion-pair formation. Thus, ammonium acetate was selected as the additive for the mobile phase, and the amount and pH were selected according to (Shibata et al., 2013)).

1.2. Specificity

The method clearly separates ACBI2, cholesterol, DOPG, DSPE-mPEG_2000_, and DOPC (Figure 2). It also separates common lipid degradation products such as 18:1 LPC, 18:0 LPC, oleic acid, and stearic acid. To test for interference of degradation products with other analytes and to elucidate degradation pathways, the formulation components were subjected to harsh conditions by incubating them in 0.5 M HCl, 0.5 M NaOH, and 3% H_2_O_2_ for 2 h at room temperature before HPLC analysis. Chromatograms illustrating the degradation pathways are shown in Figure A.1-A.3, and an overlaid chromatogram of all lipid degradation is shown in Figure A.4.

Figure A.1: Degradation profile of ACBI2: the sample was incubated for 2 h in 0.5 M HCl, 0.5 M NaOH, and 3% H_2_O_2_. The resulting chromatograms of the CAD and DAD are displayed here and show that degradation mainly occurred with 3% H_2_O_2_.

Figure A.2: Degradation profile of ACBI3: the API was incubated for 2 h in 0.5 M HCl, 0.5 M NaOH, and 3% H_2_O_2_. The resulting chromatograms of the CAD and DAD are displayed here and show that degradation mainly occurred with 3% H_2_O_2_, and there was no interference of the degradation products with the main peak.

Figure A.3: Degradation profile of the lipids. All lipids were incubated for 2 h in 0.5 M HCl, 0.5 M NaOH, and 3% H_2_O_2_. The resulting chromatograms of CAD and DAD are shown (from top to bottom: DOPC, DOPG, cholesterol, and DSPE-mPEG_2000_). Degradation products are mainly the respective lysolipids and fatty acids. Cholesterol remained stable under these conditions.

ACBI2 and ACBI3 were mainly degraded by H_2_O_2_, and no degradation products interfered with the analysis of other components. As expected, the most common degradation products of DOPC were 18:1 LPC and oleic acid. The only degradation product of DOPG that could be detected was oleic acid. Cholesterol remained stable under these conditions. DSPE-mPEG_2000_ was degraded to stearic acid and further non-identified degradation products (Figure A.4).

Figure A.4: Overlaid chromatograms of all lipids after forced degradation by incubation in 0.5 M HCl, 0.5 M NaOH, and 3% H_2_O_2_. No degradation peak interfered with the sample peaks, and most degradation peaks could be identified.

1.3. Calibration – working range

The components were calibrated according to their possible proportions in the formulations: ACBI2 was calibrated for a range of 10–600 µM, ACBI3 for 10–450 µM, and DSPE-mPEG_2000_ for 10–450 µM. The main lipids (cholesterol, DOPG, and DOPC) were calibrated for a range of 10–1250 µM, and the degradation products 18:0 LPC, 18:1 LPC, and oleic acid were calibrated for 10–450 µM. The resulting calibration plots are presented in Figure A.5. ACBI2 and ACBI3 were quantified using DAD detection because it was more sensitive and specific, and calibration was performed using a linear fit. Charged aerosol detection does not result in linear calibration plots when higher sample concentrations (Vervoort et al., 2008). Therefore, a second-order polynomial fit was selected, as suggested in the literature (Bender et al., 2024; Boßmann et al., 2019; Langer and Süss, 2021; Weber et al., 2020). High R^2^ values (> 0.99) confirmed the successful calibration.

Figure A.5: Calibration plots of all components. The fit was applied according to the detector used (linear fit for ACBI2 and ACBI3; otherwise, second-order polynomial). The equations of the components and their coefficients of determination are displayed in the graphs.

The lower detection (LOD) and quantitation (LOQ) limits are fixed by the ICH Guideline Q2(R2) to a signal-to-noise (S/N) ratio of 3 and 10, respectively (European Medicines Agency (EMA), 2023). These lower limits were calculated based on the S/N ratio derived from the Chromeleon software for samples with known low concentrations, and the results are shown in Table A.2. To better understand the quantitation limits, the LOQ values were converted to mass concentrations, as charged aerosol detection is mass-dependent. LOQ values were very low (< 5.1 µg mL^-1^) for ACBI2, ACBI3, cholesterol, DOPG, DOPC, and 18:1 LPC. The quantitation limits of cholesterol, DSPE-mPEG_2000_, oleic acid, and 18:0 LPC were higher (up to 42.69  µg mL^-1^). The high LOQ of DSPE-mPEG_2000_ can be explained by its broad peak originating from its mass range. Oleic acid and 18:0 LPC are degradation products with lower molecular weights and, consequently, weaker signals, and the baseline surrounding these peaks is less even than in other areas of the chromatogram, resulting in a higher S/N ratio. The lower limits were found to be sufficient for the intended purpose of the method.

Table A.2: Limit of Detection (LOD) and limit of quantitation (LOQ) of all components. The values were calculated based on the signal-to-noise ratio of calibration samples with low concentrations.

| Component | LOD (µM) | LOQ (µM) | LOQ (µg mL^-1^) |
| --- | --- | --- | --- |
| ACBI2 | 1.42 | 4.73 | 5.04 |
| ACBI3 | 0.44 | 1.47 | 1.50 |
| Cholesterol | 3.77 | 12.55 | 4.85 |
| DOPG | 0.95 | 3.16 | 2.52 |
| DOPC | 0.42 | 1.40 | 1.10 |
| DSPE-mPEG_2000_ | 4.56 | 15.22 | 42.69 |
| Oleic Acid | 12.45 | 41.52 | 11.73 |
| 18:1 LPC | 1.55 | 5.15 | 2.69 |
| 18:0 LPC | 10.02 | 33.38 | 17.48 |

1.4. Precision

Precision was estimated to determine the variations in retention time and area originating from the HPLC system, method, and eluent preparation. It was measured by analyzing three different concentrations of each component. For repeatability, each sample was injected three times on the same day. Intermediate precision was determined through the analysis of the same sample on three different days. Peak results were analyzed with respect to their retention time and peak area. The results for the retention times are shown in Table A.3, and the results for the peak areas are displayed in Table A.4 (DAD detection) and A.5 (CAD detection).

The retention times of all components were highly precise in terms of repeatability and intermediate precision, as indicated by relative standard deviations (RSDs) of ≤ 0.53%.

The repeatability of the areas was excellent, as demonstrated by RSDs of less than 1.1%. Intermediate precision was lower but satisfactory. The signal produced by the diode array detector was more precise than that of the CAD, as reflected by RSDs ≤ 1.53% (DAD) in contrast to RSDs of up to 4.02% (CAD). As this lower precision was obtained for formulation components and not for the API itself, and similar precisions have been reported using charged aerosol detection, this precision was found to be acceptable (Kinsey et al., 2022; Mousli et al., 2022).

Table A.3: Repeatability and intermediate precision of the retention times of all components. ACBI2, ACBI3, DSPE-mPEG_2000_, oleic acid, 18:1 LPC, and 18:0 LPC were analyzed at concentrations of 50, 100, and 200 µM. The main lipids (DOPC, DOPG, and cholesterol) were analyzed at concentrations of 300, 600, and 900 µM, respectively. Repeatability represents variability within the same day, and intermediate precision was analyzed for inter-day variability. Data are shown as mean ± SD, relative standard deviation (RSD), and mean ± confidence intervals (CI) (α=5%); (n=3).

| Component | Concentration (µM) | Repeatability | | |  | Intermediate Precision | | |
| --- | --- | --- | --- | --- | --- | --- | --- | --- |
|  |  | Average ± SD (min) | RSD (%) | CI (95%) (min) |  | Average ± SD (min) | RSD (%) | CI (95%) (min) |
| ACBI2 | 50 | 7.18 ± 0.00 | 0.02 | 7.18 ± 0.00 |  | 7.19 ± 0.02 | 0.21 | 7.19 ± 0.04 |
|  | 100 | 7.16 ± 0.00 | 0.02 | 7.16 ± 0.00 |  | 7.18 ± 0.01 | 0.20 | 7.18 ± 0.01 |
|  | 200 | 7.19 ± 0.00 | 0.00 | 7.19 ± 0.00 |  | 7.20 ± 0.02 | 0.29 | 7.20 ± 0.05 |
| ACBI3 | 50 | 6.58 ± 0.00 | 0.00 | 6.58 ± 0.00 |  | 6.57 ± 0.01 | 0.15 | 6.57 ± 0.02 |
|  | 100 | 6.58 ± 0.00 | 0.00 | 6.58 ± 0.00 |  | 6.57 ± 0.01 | 0.18 | 6.57 ± 0.03 |
|  | 200 | 6.58 ± 0.00 | 0.03 | 6.58 ± 0.00 |  | 6.57 ± 0.01 | 0.15 | 6.57 ± 0.02 |
| Cholesterol | 300 | 30.18 ± 0.02 | 0.08 | 30.18 ± 0.06 |  | 30.25 ± 0.06 | 0.20 | 30.25 ± 0.15 |
|  | 600 | 30.26 ± 0.03 | 0.11 | 30.26 ± 0.08 |  | 30.22 ± 0.12 | 0.40 | 30.22 ± 0.30 |
|  | 900 | 30.27 ± 0.02 | 0.06 | 30.27 ± 0.05 |  | 30.14 ± 0.22 | 0.74 | 30.14 ± 0.56 |
| DOPG | 300 | 35.90 ± 0.02 | 0.07 | 35.90 ± 0.06 |  | 35.88 ± 0.06 | 0.17 | 35.88 ± 0.16 |
|  | 600 | 35.81 ± 0.00 | 0.01 | 35.81 ± 0.01 |  | 35.81 ± 0.08 | 0.21 | 35.81 ± 0.19 |
|  | 900 | 35.72 ± 0.01 | 0.04 | 35.72 ± 0.03 |  | 35.73 ± 0.09 | 0.24 | 35.73 ± 0.21 |
| DOPC | 300 | 41.24 ± 0.01 | 0.03 | 41.24 ± 0.04 |  | 41.21 ± 0.08 | 0.19 | 41.21 ± 0.19 |
|  | 600 | 41.18 ± 0.01 | 0.01 | 41.18 ± 0.02 |  | 41.16 ± 0.05 | 0.11 | 41.16 ± 0.12 |
|  | 900 | 41.17 ± 0.01 | 0.02 | 41.17 ± 0.02 |  | 41.16 ± 0.03 | 0.06 | 41.16 ± 0.06 |
| DSPE-mPEG_2000_ | 50 | 38.61 ± 0.04 | 0.10 | 38.61 ± 0.09 |  | 38.52 ± 0.08 | 0.20 | 38.52 ± 0.19 |
|  | 100 | 38.63 ± 0.01 | 0.02 | 38.63 ± 0.02 |  | 38.54 ± 0.08 | 0.20 | 38.54 ± 0.19 |
|  | 200 | 38.56 ± 0.01 | 0.04 | 38.56 ± 0.04 |  | 38.49 ± 0.07 | 0.18 | 38.49 ± 0.17 |
| Oleic Acid | 50 | 11.33 ± 0.00 | 0.02 | 11.33 ± 0.01 |  | 11.31 ± 0.06 | 0.53 | 11.31 ± 0.15 |
|  | 100 | 11.33 ± 0.00 | 0.04 | 11.33 ± 0.01 |  | 11.31 ± 0.06 | 0.51 | 11.31 ± 0.14 |
|  | 200 | 11.33 ± 0.01 | 0.08 | 11.33 ± 0.02 |  | 11.31 ± 0.05 | 0.46 | 11.31 ± 0.13 |
| 18:1 LPC | 50 | 9.86 ± 0.01 | 0.11 | 9.86 ± 0.03 |  | 9.86 ± 0.00 | 0.05 | 9.86 ± 0.01 |
|  | 100 | 9.85 ± 0.00 | 0.04 | 9.85 ± 0.01 |  | 9.85 ± 0.01 | 0.07 | 9.85 ± 0.02 |
|  | 200 | 9.84 ± 0.00 | 0.05 | 9.84 ± 0.01 |  | 9.84 ± 0.00 | 0.04 | 9.84 ± 0.01 |
| 18:0 LPC | 50 | 11.12 ± 0.00 | 0.04 | 11.12 ± 0.01 |  | 11.11 ± 0.01 | 0.08 | 11.11 ± 0.02 |
|  | 100 | 11.11 ± 0.01 | 0.06 | 11.11 ± 0.02 |  | 11.10 ± 0.01 | 0.11 | 11.10 ± 0.03 |
|  | 200 | 11.09 ± 0.00 | 0.02 | 11.09 ± 0.01 |  | 11.08 ± 0.00 | 0.04 | 11.08 ± 0.01 |

Table A.4: Repeatability and intermediate precision of the areas of ACBI2 and ACBI3. Both were analyzed at concentrations of 50, 100, and 200 µM. Repeatability represents variability within the same day, and intermediate precision was analyzed for inter-day variability. Data are shown as mean ± SD, relative standard deviation (RSD), and mean ± confidence intervals (CI) (α=5%); (n=3).

| Component | Concentration (µM) | Repeatability | | |  | Intermediate Precision | | |
| --- | --- | --- | --- | --- | --- | --- | --- | --- |
|  |  | Average ± SD (mAU min) | RSD (%) | CI (95%) (mAU min) |  | Average ± SD (mAU min) | RSD (%) | CI (95%) (mAU min) |
| ACBI2 | 50 | 24.52 ± 0.06 | 0.24 | 24.52 ± 0.15 |  | 24.81 ± 0.25 | 1.01 | 24.81 ± 0.62 |
|  | 100 | 48.27 ± 0.01 | 0.03 | 48.27 ± 0.04 |  | 48.50 ± 0.33 | 0.68 | 48.50 ± 0.25 |
|  | 200 | 97.69 ± 0.01 | 0.01 | 97.69 ± 0.03 |  | 97.48 ± 0.83 | 0.85 | 97.48 ± 2.05 |
| ACBI3 | 50 | 11.32 ± 0.06 | 0.51 | 11.32 ± 0.14 |  | 11.44 ± 0.15 | 1.33 | 11.44 ± 0.38 |
|  | 100 | 22.98 ± 0.11 | 0.48 | 22.98 ± 0.28 |  | 23.10 ± 0.16 | 0.70 | 23.10 ± 0.40 |
|  | 200 | 45.81 ± 0.10 | 0.21 | 45.81 ± 0.24 |  | 46.62 ± 0.71 | 1.53 | 46.62 ± 1.77 |

Table A.5: Repeatability and intermediate precision of the areas of all lipid components. The main lipids (DOPC, DOPG, and cholesterol) were analyzed at concentrations of 300 µM, 600 µM, and 900 µM, and DSPE-mPEG_2000_, oleic acid, 18:1 LPC, and 18:0 LPC were analyzed at concentrations of 50 µM, 100 µM, and 200 µM. Repeatability represents variability within the same day, and intermediate precision was analyzed for inter-day variability. Data are shown as mean ± SD, relative standard deviation (RSD), and mean ± confidence intervals (CI) (α=5%); (n=3).

| Component | Concentration (µM) | Repeatability | | | | Intermediate Precision | | | |
| --- | --- | --- | --- | --- | --- | --- | --- | --- | --- |
|  |  | Average ± SD (pA min) | RSD (%) | CI (95%) (pA min) |  | | Average ± SD (pA min) | RSD (%) | CI (95%) (pA min) |
| Cholesterol | 300 | 13.76 ± 0.03 | 0.20 | 13.76 ± 0.07 |  | | 13.30 ± 0.52 | 3.88 | 13.30 ± 1.28 |
|  | 600 | 26.86 ± 0.28 | 1.05 | 26.86 ± 0.70 |  | | 26.39 ± 1.06 | 4.02 | 26.39 ± 2.64 |
|  | 900 | 39.06 ± 0.17 | 0.43 | 39.06 ± 0.42 |  | | 38.20 ± 0.89 | 2.34 | 38.20 ± 2.22 |
| DOPG | 300 | 27.64 ± 0.16 | 0.59 | 27.64 ± 0.40 |  | | 27.90 ± 0.93 | 3.34 | 27.90 ± 2.32 |
|  | 600 | 50.86 ± 0.12 | 0.24 | 50.86 ± 0.31 |  | | 51.31 ± 1.56 | 3.05 | 51.31 ± 3.89 |
|  | 900 | 72.58 ± 0.07 | 0.10 | 72.58 ± 0.17 |  | | 73.37 ± 1.81 | 2.46 | 73.37 ± 4.49 |
| DOPC | 300 | 31.64 ± 0.04 | 0.12 | 31.64 ± 0.09 |  | | 31.99 ± 0.90 | 2.81 | 31.99 ± 2.23 |
|  | 600 | 56.28 ± 0.18 | 0.32 | 56.28 ± 0.44 |  | | 57.00 ± 1.36 | 2.39 | 57.00 ± 3.38 |
|  | 900 | 77.51 ± 0.10 | 0.13 | 77.51 ± 0.24 |  | | 78.47 ± 1.79 | 2.28 | 78.47 ± 4.44 |
| DSPE-mPEG_2000_ | 50 | 20.69 ± 0.06 | 0.31 | 20.69 ± 0.16 |  | | 20.70 ± 0.25 | 1.19 | 20.70 ± 0.61 |
|  | 100 | 39.91 ± 0.40 | 1.01 | 39.91 ± 1.00 |  | | 39.95 ± 0.33 | 0.82 | 39.95 ± 0.81 |
|  | 200 | 74.39 ± 0.32 | 0.43 | 74.39 ± 0.80 |  | | 74.49 ± 0.29 | 0.39 | 74.49 ± 0.72 |
| Oleic Acid | 50 | 0.84 ± 0.01 | 0.78 | 0.84 ± 0.02 |  | | 0.87 ± 0.02 | 2.60 | 0.87 ± 0.06 |
|  | 100 | 1.49 ± 0.00 | 0.09 | 1.49 ± 0.00 |  | | 1.55 ± 0.06 | 3.84 | 1.55 ± 0.15 |
|  | 200 | 2.91 ± 0.02 | 0.70 | 2.91 ± 0.05 |  | | 2.97 ± 0.06 | 1.95 | 2.97 ± 0.14 |
| 18:1 LPC | 50 | 2.88 ± 0.01 | 0.26 | 2.88 ± 0.02 |  | | 2.89 ± 0.04 | 1.28 | 2.89 ± 0.09 |
|  | 100 | 5.87 ± 0.02 | 0.35 | 5.87 ± 0.05 |  | | 5.85 ± 0.06 | 1.00 | 5.85 ± 0.14 |
|  | 200 | 11.55 ± 0.02 | 0.19 | 11.55 ± 0.06 |  | | 11.49 ± 0.12 | 1.03 | 11.49 ± 0.29 |
| 18:0 LPC | 50 | 2.98 ± 0.03 | 0.86 | 2.98 ± 0.06 |  | | 2.96 ± 0.04 | 1.21 | 2.96 ± 0.09 |
|  | 100 | 6.00 ± 0.04 | 0.64 | 6.00 ± 0.10 |  | | 5.93 ± 0.11 | 1.77 | 5.93 ± 0.26 |
|  | 200 | 11.60 ± 0.02 | 0.17 | 11.60 ± 0.05 |  | | 11.68 ± 0.07 | 0.61 | 11.68 ± 0.18 |

1.5. Accuracy

Accuracy is defined as the detected concentration relative to the anticipated concentration. The analysis was performed in triplicate using three separately prepared stock solutions. The accuracy of each component was analyzed at three different concentration levels (Table A.6). We achieved high accuracy for all components (97.43-101.67%).

Table A.6: Accuracy of all components. ACBI2, ACBI3, DSPE-mPEG_2000_, oleic acid, 18:1 LPC, and 18:0 LPC were analyzed at concentration levels of 50 µM, 100 µM, and 200 µM. Main lipids (DOPC, DOPG, and cholesterol) were analyzed in concentrations of 300 µM, 600 µM, and 900 µM. Accuracy was calculated as analyzed concentration relative to the expected concentration. Data is shown as mean ± SD, relative standard deviation (RSD), and mean ± confidence intervals (CI) (α=5%); (n=3).

| Component | Concentration (µM) | Mean % Recovery ± SD | Mean % Recovery ± CI (95%) |
| --- | --- | --- | --- |
| ACBI2 | 50 | 98.79 ± 2.28 | 98.79 ± 5.66 |
|  | 100 | 99.96 ± 1.35 | 99.96 ± 3.37 |
|  | 200 | 99.91 ± 1.26 | 99.91 ± 3.13 |
| ACBI3 | 50 | 101.16 ± 0.25 | 101.16 ± 0.62 |
|  | 100 | 99.76 ± 0.42 | 99.76 ± 1.05 |
|  | 200 | 99.59 ± 0.42 | 99.59 ± 1.05 |
| Cholesterol | 300 | 101.32 ± 1.32 | 101.32 ± 3.28 |
|  | 600 | 99.98 ± 1.05 | 99.98 ± 2.61 |
|  | 900 | 100.68 ± 0.87 | 100.68 ± 2.17 |
| DOPG | 300 | 100.03 ± 2.59 | 100.03 ± 6.43 |
|  | 600 | 99.71 ± 1.98 | 99.71 ± 4.92 |
|  | 900 | 100.35 ± 0.41 | 100.35 ± 1.01 |
| DOPC | 300 | 98.13 ± 0.99 | 98.13 ± 2.47 |
|  | 600 | 101.67 ± 2.50 | 101.67 ± 6.21 |
|  | 900 | 101.41 ± 1.18 | 101.41 ± 2.94 |
| DSPE-mPEG_2000_ | 50 | 99.68 ± 5.12 | 99.68 ± 12.72 |
|  | 100 | 99.62 ± 3.59 | 99.62 ± 8.91 |
|  | 200 | 99.67 ± 2.68 | 99.67 ± 6.66 |
| Oleic Acid | 50 | 97.43 ± 4.45 | 97.43 ± 11.04 |
|  | 100 | 97.85 ± 4.53 | 97.85 ± 11.26 |
|  | 200 | 98.65 ± 3.98 | 98.65 ± 9.88 |
| 18:1 LPC | 50 | 99.75 ± 0.95 | 99.75 ± 2.36 |
|  | 100 | 100.26 ± 2.50 | 100.26 ± 6.21 |
|  | 200 | 99.36 ± 2.35 | 99.36 ± 5.83 |
| 18:0 LPC | 50 | 100.27 ± 2.00 | 100.27 ± 4.96 |
|  | 100 | 100.04 ± 1.10 | 100.04 ± 2.73 |
|  | 200 | 100.94 ± 0.42 | 100.94 ± 1.05 |

1.6. Recovery of dual centrifugation-manufactured ACBI2-liposomes

Finally, we prepared two different liposomal formulations containing ACBI2, cholesterol, DOPC, DOPG, and DSPE-mPEG_2000_ using dual centrifugation and analyzed the recovery of each component before and after purification *via* centrifugation. The experiment was performed in triplicate. Details on the formulations and the recovery rates are provided in Table A.7. The recovery of ACBI2 after purification equals the incorporation efficiency. We achieved high recovery rates (> 87%) for each component prior to purification. The loss of lipids during centrifugation can be attributed to lipids adhering to the vials and lipid residues inside the micro tubes for dual centrifugation. This phenomenon is particularly pronounced for cholesterol and has been previously described (Bender et al., 2024). We also investigated lipid degradation originating from dual centrifugation and detected no degradation products (Figure A.6). Ultimately, we also validated our purification approach with samples containing only ACBI2 in histidine buffer (Table A.8).

Table A.7: Recovery of liposomal ACBI2 formulations prepared by dual centrifugation before and after purification via centrifugation (10,000 × g, 10 min, 22 °C). The experiment was conducted three times, and each sample was analyzed in triplicate. Data are presented as mean ± SD.

| Formulation mixture | ACBI2 (%) | Cholesterol (%) | DOPG (%) | DOPC (%) | DSPE-mPEG_2000_ (%) |
| --- | --- | --- | --- | --- | --- |
| Formulation 1  DOPC / DOPG / DSPE-mPEG_2000_  50 / 45 / 5  + 6% (*n/n*) ACBI2 | 91.38 ± 2.71 |  | 98.46 ± 1.86 | 104.32 ± 1.87 | 99.8 ± 2.56 |
| Purified: | 90.89 ± 5.74 |  | 94.77 ± 3.88 | 101.52 ± 6.73 | 95.55 ± 3.49 |
| Formulation 2  DOPC / DOPG / Cholesterol  40 / 40 / 20  + 6% (*n/n*) ACBI2 | 87.83 ± 2.31 | 90.11 ± 0.93 | 96.45 ± 2.14 | 97.17 ± 3.24 |  |
| Purified: | 72.97 ± 5.18 | 85.53 ± 1.90 | 89.99 ± 0.62 | 91.81 ± 1.58 |  |

Table A.8: Validation of purification procedure. HPLC analysis of samples containing only 1.2 mg ACBI2 in histidine buffer after two cycles of dual centrifugation at 1000 rpm and 10 °C for 1 min and subsequent centrifugation at 5,000 x g and 22 °C for 10 min. The recovered amount of ACBI2 in each fraction (supernatant, pellet, and residue in vial for dual centrifugation) is shown, as well as the signal-to-noise (S/N) ratio. At the API concentrations and sample dilutions employed for the liposomes, the non-pelletized amount of ACBI2 is below the limit of quantification. Data are presented as mean ± SD, n = 3.

|  | % ACBI2 of total (n/n) | S/N-ratio |
| --- | --- | --- |
| supernatant | 0.98 ± 0.90 | 6.97 ± 2.61 |
| pellet | 80.24 ± 1.91 | 786.83 ± 72.35 |
| residue in DC vial | 18.78 ± 1.43 | 1507.13 ± 912.91 |

Figure A.6: Chromatograms of liposomal ACBI2 formulations produced via dual centrifugation. The upper chromatogram shows formulation 1 (DOPC/DOPG/DSPE-mPEG_2000_ 50/45/5 + 6% (n/n) ACBI2) the lower one formulation 2 (DOPC/DOPG/Cholesterol 40/40/20 + 6% (n/n) ACBI2) No degradation occurred during dual centrifugation.

2. Design of Experiments (DoE)

Table A.9: Experimental Plan. This table contains all information on the design points of the DoE approach, including batch number, run number, type of design point, lipid composition, pH, and lipid concentration during dual centrifugation. The DoE was conducted strictly following this plan.

|  |  |  | Component 1 | Component 2 | Component 3 | Component 4 | Factor 5 | Factor 6 |
| --- | --- | --- | --- | --- | --- | --- | --- | --- |
| Block | Run | Build Type | A:DOPC | B:DOPG | C:Cholesterol | D:DSPE-mPEG_2000_ | E:pH | F:lipid concentration |
|  |  |  | % | % | % | % |  | % |
| batch1 | 1 | Model | 95 | 0 | 0 | 5 | 7 | 60 |
| batch1 | 2 | Model | 50 | 50 | 0 | 0 | 5 | 60 |
| batch1 | 3 | Model | 100 | 0 | 0 | 0 | 6 | 20 |
| batch1 | 4 | Model | 45 | 50 | 0 | 5 | 6 | 40 |
| batch1 | 5 | Model | 20 | 30 | 45 | 5 | 5 | 60 |
| batch1 | 6 | Replicate | 20 | 50 | 30 | 0 | 7 | 60 |
| batch1 | 7 | Model | 55 | 0 | 45 | 0 | 7 | 20 |
| batch1 | 8 | Model | 75 | 0 | 22.5 | 2.5 | 7 | 40 |
| batch1 | 9 | Model | 50 | 50 | 0 | 0 | 7 | 20 |
| batch1 | 10 | Model | 20 | 50 | 25 | 5 | 7 | 20 |
| batch1 | 11 | Model | 20 | 50 | 25 | 5 | 5 | 20 |
| batch1 | 12 | Model | 100 | 0 | 0 | 0 | 5 | 60 |
| batch1 | 13 | Model | 50 | 0 | 45 | 5 | 7 | 60 |
| batch1 | 14 | Replicate | 50 | 50 | 0 | 0 | 5 | 60 |
| batch1 | 15 | Model | 70 | 25 | 0 | 5 | 5 | 20 |
| batch1 | 16 | Model | 60 | 25.75 | 11.75 | 2.5 | 6 | 60 |
| batch1 | 17 | Model | 52.5 | 0 | 45 | 2.5 | 5 | 40 |
| batch1 | 18 | Model | 20 | 50 | 30 | 0 | 7 | 60 |
| batch1 | 19 | Lack of Fit | 100 | 0 | 0 | 0 | 6 | 40 |
| batch1 | 20 | Model | 20 | 35 | 45 | 0 | 5 | 20 |
| batch2 | 21 | Model | 20 | 50 | 30 | 0 | 6 | 40 |
| batch2 | 22 | Model | 100 | 0 | 0 | 0 | 5 | 20 |
| batch2 | 23 | Lack of Fit | 50 | 50 | 0 | 0 | 6 | 20 |
| batch2 | 24 | Lack of Fit | 45 | 50 | 0 | 5 | 5 | 40 |
| batch2 | 25 | Model | 50 | 0 | 45 | 5 | 5 | 20 |
| batch2 | 26 | Model | 95 | 0 | 0 | 5 | 7 | 20 |
| batch2 | 27 | Model | 95 | 0 | 0 | 5 | 5 | 60 |
| batch2 | 28 | Model | 55 | 0 | 45 | 0 | 7 | 60 |
| batch2 | 29 | Model | 20 | 50 | 25 | 5 | 5 | 60 |
| batch2 | 30 | Replicate | 95 | 0 | 0 | 5 | 5 | 60 |
| batch2 | 31 | Model | 55 | 0 | 45 | 0 | 5 | 60 |
| batch2 | 32 | Replicate | 95 | 0 | 0 | 5 | 7 | 20 |
| batch2 | 33 | Model | 75 | 25 | 0 | 0 | 7 | 40 |
| batch2 | 34 | Model | 49 | 27 | 24 | 0 | 5 | 20 |
| batch2 | 35 | Model | 100 | 0 | 0 | 0 | 7 | 60 |
| batch2 | 36 | Model | 50 | 0 | 45 | 5 | 6 | 20 |
| batch2 | 37 | Model | 45 | 50 | 0 | 5 | 7 | 60 |
| batch2 | 38 | Model | 20 | 30 | 45 | 5 | 7 | 40 |
| batch2 | 39 | Model | 47.5 | 50 | 0 | 2.5 | 5 | 20 |
| batch2 | 40 | Model | 20 | 32.5 | 45 | 2.5 | 7 | 20 |

Table A.10: Results obtained from the experimental plan. Each experiment was carried out according to the experimental plan, and the individual responses are shown here. Run 28 was excluded from the analysis because a very inhomogeneous mixture resulted from the formulation, which had no liposomes and, therefore, could not be analyzed correctly.

|  | Response 1 | Response 2 | Response 3 | Response 4 | Response 5 |
| --- | --- | --- | --- | --- | --- |
| Run | Z-Average  (nm) | PDI | Zeta Potential (mV) | D/L-Ratio  (%) | IE  (%) |
| 1 | 158.8 | 0.193 | -3.6 | 3.6 | 63.6 |
| 2 | 198.8 | 0.453 | -73.5 | 5.1 | 80.0 |
| 3 | 221.8 | 0.382 | 10.2 | 5.5 | 86.4 |
| 4 | 95.3 | 0.191 | -9.0 | 5.7 | 90.8 |
| 5 | 138.2 | 0.219 | -12.3 | 3.0 | 47.2 |
| 6 | 229.3 | 0.120 | -72.5 | 4.9 | 73.2 |
| 7 | 146.5 | 0.186 | -4.7 | 1.0 | 8.8 |
| 8 | 118.2 | 0.201 | -3.7 | 2.4 | 36.8 |
| 9 | 75.8 | 0.180 | -22.9 | 5.8 | 86.0 |
| 10 | 92.3 | 0.227 | -8.4 | 5.9 | 76.8 |
| 11 | 88.1 | 0.211 | -7.8 | 6.1 | 87.6 |
| 12 | 329.3 | 0.232 | 11.5 | 3.6 | 64.4 |
| 13 | 168.8 | 0.300 | -5.9 | 1.4 | 12.8 |
| 14 | 178.9 | 0.345 | -73.9 | 5.7 | 83.2 |
| 15 | 103.7 | 0.182 | -10.1 | 5.8 | 88.8 |
| 16 | 148.1 | 0.199 | -22.0 | 5.3 | 86.4 |
| 17 | 104.2 | 0.231 | -2.9 | 2.3 | 21.2 |
| 18 | 221.1 | 0.083 | -73.0 | 5.2 | 78.4 |
| 19 | 224.8 | 0.140 | 20.5 | 5.1 | 84.0 |
| 20 | 94.0 | 0.132 | -41.6 | 4.4 | 63.2 |
| 21 | 149.6 | 0.07 | -65.5 | 4.0 | 57.2 |
| 22 | 173.7 | 0.247 | 12.5 | 4.4 | 68.0 |
| 23 | 83.4 | 0.157 | -34.8 | 5.1 | 71.2 |
| 24 | 101.0 | 0.188 | -10.5 | 5.4 | 81.6 |
| 25 | 103.3 | 0.227 | -3.0 | 3.0 | 27.2 |
| 26 | 117.3 | 0.209 | -4.7 | 4.3 | 64.4 |
| 27 | 157.5 | 0.202 | -0.7 | 4.6 | 77.6 |
| 28 | 1667.5 | 0.880 | -2.2 | 2.3 | 2.4 |
| 29 | 139.0 | 0.234 | -16.1 | 3.5 | 52.0 |
| 30 | 257.9 | 0.161 | 12.7 | 3.4 | 57.6 |
| 31 | 228.3 | 0.162 | 2.1 | 1.9 | 10.0 |
| 32 | 121.3 | 0.215 | -3.0 | 4.3 | 67.6 |
| 33 | 220.6 | 0.190 | -49.9 | 4.8 | 75.6 |
| 34 | 112.2 | 0.136 | -34.1 | 5.8 | 79.2 |
| 35 | 280.2 | 0.223 | 3.6 | 3.8 | 36.4 |
| 36 | 100.5 | 0.215 | -4.1 | 3.4 | 32.4 |
| 37 | 122.1 | 0.168 | -14.3 | 5.5 | 84.4 |
| 38 | 95.8 | 0.192 | -8.0 | 4.8 | 68.8 |
| 39 | 108.3 | 0.191 | -16.5 | 5.5 | 81.2 |
| 40 | 89.9 | 0.233 | -13.4 | 3.1 | 46.0 |

Table A.11: ANOVA results of the DoE-based response models as computed by Stat-Ease 360. Each response is reported, comprising data transformation, included terms, sum of squares, degrees of freedom (df), mean square, F-value, and p-value.

| **Response** | **Source** | **Sum of Squares** | **df** | **Mean Square** | **F-value** | **p-value** |  |
| --- | --- | --- | --- | --- | --- | --- | --- |
| **Z-Average** | Block | 0.0001 | 1 | 0.0001 |  |  |  |
| Transform: | **Model** | **0.0079** | **7** | **0.0011** | **21.92** | **< 0.0001** | **significant** |
| $\boldsymbol{y}^{\boldsymbol{'}}\boldsymbol{=}\boldsymbol{1}\boldsymbol{/}\sqrt{\boldsymbol{y}}$ | Linear Mixture | 0.0035 | 3 | 0.0012 | 22.31 | < 0.0001 |  |
|  | AF | 0.0006 | 1 | 0.0006 | 11.87 | 0.0017 |  |
|  | BC | 0.0003 | 1 | 0.0003 | 6.48 | 0.0163 |  |
|  | BF | 0.0012 | 1 | 0.0012 | 22.93 | < 0.0001 |  |
|  | CF | 0.0003 | 1 | 0.0003 | 5.72 | 0.0232 |  |
|  | **Residual** | 0.0016 | 30 | 0.0001 |  |  |  |
|  | Lack of Fit | 0.0014 | 26 | 0.0001 | 1.33 | 0.4348 | not significant |
|  | Pure Error | 0.0002 | 4 | 0.0000 |  |  |  |
|  | **Cor Total** | 0.0095 | 38 |  |  |  |  |
| **PDI** | Block | 0.1041 | 1 | 0.1041 |  |  |  |
| Transform: | **Model** | **2.85** | **8** | **0.3568** | **6.59** | **< 0.0001** | **significant** |
| $\boldsymbol{y}^{\boldsymbol{'}}\boldsymbol{=}\ln\boldsymbol{y}$ | Linear Mixture | 0.664 | 3 | 0.2213 | 4.09 | 0.0154 |  |
|  | BC | 0.242 | 1 | 0.242 | 4.47 | 0.0432 |  |
|  | BE | 0.2409 | 1 | 0.2409 | 4.45 | 0.0436 |  |
|  | CD | 0.9405 | 1 | 0.9405 | 17.38 | 0.0003 |  |
|  | EF | 0.2929 | 1 | 0.2929 | 5.41 | 0.0272 |  |
|  | F² | 0.2329 | 1 | 0.2329 | 4.3 | 0.047 |  |
|  | **Residual** | 1.57 | 29 | 0.0541 |  |  |  |
|  | Lack of Fit | 1.44 | 25 | 0.0575 | 1.75 | 0.3133 | not significant |
|  | Pure Error | 0.1312 | 4 | 0.0328 |  |  |  |
|  | **Cor Total** | 4.53 | 38 |  |  |  |  |
| **Zeta Potential** | Block | 1.24E+05 | 1 | 1.24E+05 |  |  |  |
| Transform: | **Model** | **1.02E+07** | **9** | **1.14E+06** | **43.06** | **< 0.0001** | **significant** |
| Power | Linear Mixture | 6.78E+06 | 3 | 2.26E+06 | 85.67 | < 0.0001 |  |
| $\boldsymbol{y}^{\boldsymbol{'}}\boldsymbol{=}{\boldsymbol{(}\boldsymbol{y}\boldsymbol{+}\boldsymbol{81}\boldsymbol{.}\boldsymbol{98}\boldsymbol{)}}^{\boldsymbol{1}\boldsymbol{.}\boldsymbol{65}}$ | AB | 3.89E+05 | 1 | 3.89E+05 | 14.76 | 0.0006 |  |
|  | AC | 1.70E+05 | 1 | 1.70E+05 | 6.46 | 0.0168 |  |
|  | AE | 1.42E+05 | 1 | 1.42E+05 | 5.4 | 0.0277 |  |
|  | BC | 3.36E+05 | 1 | 3.36E+05 | 12.74 | 0.0013 |  |
|  | BD | 1.69E+06 | 1 | 1.69E+06 | 64.08 | < 0.0001 |  |
|  | BF | 4.92E+05 | 1 | 4.92E+05 | 18.65 | 0.0002 |  |
|  | **Residual** | 7.38E+05 | 28 | 26371.88 |  |  |  |
|  | Lack of Fit | 6.55E+05 | 24 | 27288.27 | 1.31 | 0.4415 | not significant |
|  | Pure Error | 83494.26 | 4 | 20873.56 |  |  |  |
|  | **Cor Total** | 1.11E+07 | 38 |  |  |  |  |
| **D/L-Ratio** | Block | 0.2131 | 1 | 0.2131 |  |  |  |
| Transform: | **Model** | **53.52** | **6** | **8.92** | **19.72** | **< 0.0001** | **significant** |
| No transform | Linear Mixture | 44.43 | 3 | 14.81 | 32.74 | < 0.0001 |  |
|  | BC | 2.34 | 1 | 2.34 | 5.18 | 0.03 |  |
|  | DF | 4.66 | 1 | 4.66 | 10.31 | 0.0031 |  |
|  | EF | 2.02 | 1 | 2.02 | 4.47 | 0.0427 |  |
|  | **Residual** | 14.02 | 31 | 0.4523 |  |  |  |
|  | Lack of Fit | 13.08 | 27 | 0.4843 | 2.05 | 0.2554 | not significant |
|  | Pure Error | 0.945 | 4 | 0.2362 |  |  |  |
|  | **Cor Total** | 67.76 | 38 |  |  |  |  |
| **IE** | Block | 358.32 | 1 | 358.32 |  |  |  |
| Transform: | **Model** | **18632.48** | **7** | **2661.78** | **30.53** | **< 0.0001** | **significant** |
| No transform | Linear Mixture | 15233.18 | 3 | 5077.73 | 58.23 | < 0.0001 |  |
|  | AB | 756.12 | 1 | 756.12 | 8.67 | 0.0062 |  |
|  | AE | 547.05 | 1 | 547.05 | 6.27 | 0.0179 |  |
|  | AF | 634.56 | 1 | 634.56 | 7.28 | 0.0113 |  |
|  | BC | 2155.86 | 1 | 2155.86 | 24.72 | < 0.0001 |  |
|  | **Residual** | 2615.85 | 30 | 87.2 |  |  |  |
|  | Lack of Fit | 2392.09 | 26 | 92 | 1.64 | 0.3396 | not significant |
|  | Pure Error | 223.76 | 4 | 55.94 |  |  |  |
|  | **Cor Total** | 21606.65 | 38 |  |  |  |  |

Table A.12: Actual equations of all models obtained from the DoE.

|  | 1/Sqrt(Z-Average) | ln(PDI) | (Zeta Potential + 81.98)^1.65^ | D/L-Ratio | IE |
| --- | --- | --- | --- | --- | --- |
| DOPC | 0.000828 | -0.02139 | 24.37201 | 0.076293 | 1.24389 |
| DOPG | 0.001471 | -0.00515 | 17.80901 | 0.099619 | -0.15216 |
| Cholesterol | 0.001191 | -0.03006 | 31.31541 | 0.025633 | -0.64404 |
| DSPE-mPEG2000 | 0.003405 | -0.05126 | -28.5351 | 0.330504 | 2.03073 |
| AB |  |  | -0.45973 |  | 0.019202 |
| AC |  |  | -0.40112 |  |  |
| AE |  | 0.002094 | -1.18737 | -0.0055 | -0.07388 |
| AF | -3.92E-06 | -7.1E-05 |  | -0.00083 | -0.00394 |
| BC | -7.46E-06 | -0.0002 | -0.68031 | 0.000623 | 0.033453 |
| BD |  |  | 4.05729 |  |  |
| BE |  | -0.00066 | 0.296843 | -0.0055 | 0.01847 |
| BF | -9.95E-06 | -7.1E-05 | -0.19764 | -0.00083 | 0.000985 |
| CD |  | 0.003515 |  |  |  |
| CE |  | 0.002094 | 0.296843 | -0.0055 | 0.01847 |
| CF | -5.94E-06 | -7.1E-05 |  | -0.00083 | 0.000985 |
| DE |  | 0.002094 | 0.296843 | -0.0055 | 0.01847 |
| DF |  | -7.1E-05 |  | -0.0065 | 0.000985 |
| EF |  | -0.00524 |  | 0.013761 |  |
| F² |  | 0.000482 |  |  |  |

Table A.13: Breakdown of timeline and API consumption for the implementation of the DoE.

| Day # | Tasks |
| --- | --- |
| Day 1 | Preparation of all lipid films |
| Day 2 | Preparation of liposomes (first block = 20 samples), purification, preparation of HPLC samples, DLS measurements, start of HPLC measurements |
| Day 3 | Preparation of liposomes (second block = 20 samples), purification, preparation of HPLC samples, DLS measurements, continuing HPLC measurements |
| Day 4 | Zeta Potential measurements, HPLC measurements (continued), begin of data evaluation |
| Day 5 | Data evaluation and begin of model evaluation |
| Day 8 | End of HPLC measurements, data evaluation, model evaluation, determining confirmation runs |
| Day 9 | preparation of lipid films for confirmation runs |
| Day 10 | preparation of liposomes for confirmation runs, purification, DLS, zeta measurements, HPLC samples |
| Day 11 | preparation of liposomes for confirmation runs, purification, DLS, zeta measurements, HPLC samples |
| Day 12 | preparation of liposomes for confirmation runs, purification, DLS, zeta measurements, HPLC samples |
| Day 13 | end of HPLC measurements, final evaluation |
| API required | 68 mg for 40 model points + 18 mg for confirmation runs = 86 mg ACBI2 |

Figure A.7: Normal plot of residuals of all models obtained by the DoE.

Figure A.8: Predicted vs. actual plots of all models of the DoE. The predicted and actual values are in good agreement.

Figure A.9: Model diagrams derived from the DoE: influence of the process factors. (A),(B), and (C) show the effect of pH on PDI (A), zeta potential (B), and IE (C). (D) presents the effect of lipid concentration on IE.

3. Liposome preparation

Figure A.10: Comparison of ACBI2 formulations prepared with 50 mM histidine sucrose and 20 mM histidine glucose buffer. On the left, Z-Average and incorporation efficiency are displayed. Both were slightly smaller in 20 mM histidine glucose buffer. Zeta potential and PDI remained similar (on the right). Data are shown as mean ± SD, n=3.

Figure A.11: Comparison of ACBI2 liposomes with unsaturated (18:1) lipids prepared at 10 °C and 70 °C and liposomes with saturated (18:0) lipids prepared at 70 °C. All formulations were prepared in 20 mM histidine glucose buffer. Resulting liposomes are compared with respect to their Z-Average, PDI, zeta potential, and incorporation efficiency. The physicochemical properties of liposomes produced with unsaturated lipids were similar regardless of the process temperature. Most formulations prepared with saturated lipids were larger, indicating that this difference did not originate from the process temperature. Data are shown as mean ± SD, n=3.

Table A.14: Cell viability data of A549 cells treated with 250 nM ACBI2 in DMSO and in PC/PG/cholesterol/DSPE-mPEG_2000_ 50.6/42/2.4/5 using either 18:1 or 18:0 lipids. 400 cells/well were seeded onto 96-well plate, treated for 2 h after 24 h of incubation and the assay was performed 6 days later. Neither formulation had a therapeutic benefit. Data are shown as mean ± SD, n=3; SD = standard deviation.

| PC / PG / Cholesterol / DSPE-mPEG_2000_ (%) | Lipid Type | Viability (%) | SD (%) |
| --- | --- | --- | --- |
| DMSO |  | 30.0 | 5.3 |
| 50.6 / 42.0 / 2.4 / 5.0 | 18:1 | 32.5 | 6.1 |
| 50.6 / 42.0 / 2.4 / 5.0 | 18:0 | 28.3 | 1.5 |

# 3. References

Adediran, S.A., Day, T.P., Sil, D., Kimbrell, M.R., Warshakoon, H.J., Malladi, S.S., David, S.A., 2009. Synthesis of a Highly Water-Soluble Derivative of Amphotericin B with Attenuated Proinflammatory Activity. Mol. Pharm. 6, 1582–1590. https://doi.org/10.1021/mp9001602

Atilaw, Y., Poongavanam, V., Svensson Nilsson, C., Nguyen, D., Giese, A., Meibom, D., Erdelyi, M., Kihlberg, J., 2021. Solution Conformations Shed Light on PROTAC Cell Permeability. ACS Med. Chem. Lett. 12, 107–114. https://doi.org/10.1021/acsmedchemlett.0c00556

Békés, M., Langley, D.R., Crews, C.M., 2022. PROTAC targeted protein degraders: the past is prologue. Nat. Rev. Drug Discov. 21, 181–200. https://doi.org/10.1038/s41573-021-00371-6

Bender, V., Fuchs, L., Süss, R., 2024. RP-HPLC-CAD method for the rapid analysis of lipids used in lipid nanoparticles derived from dual centrifugation. Int. J. Pharm. X 7, 100255. https://doi.org/10.1016/j.ijpx.2024.100255

Boßmann, D., Bartling, B., De Vries, I., Winkler, J., Neumann, H., Lammers, F., Beutel, S., Scheper, T., 2019. Charged aerosol detector HPLC as a characterization and quantification application of biopharmaceutically relevant polysialic acid from E. coli K1. J. Chromatogr. A 1599, 85–94. https://doi.org/10.1016/j.chroma.2019.03.069

Chen, Y., Tandon, I., Heelan, W., Wang, Y., Tang, W., Hu, Q., 2022. Proteolysis-targeting chimera (PROTAC) delivery system: advancing protein degraders towards clinical translation. Chem. Soc. Rev. 51, 5330–5350. https://doi.org/10.1039/D1CS00762A

Ermondi, G., Vallaro, M., Goetz, G., Shalaeva, M., Caron, G., 2020. Updating the portfolio of physicochemical descriptors related to permeability in the beyond the rule of 5 chemical space. Eur. J. Pharm. Sci. 146, 105274. https://doi.org/10.1016/j.ejps.2020.105274

European Medicines Agency (EMA), 2023. ICH Q2(R2) Guideline on validation of analytical procedures.

FDA, 2012. NDA 203985 Review - Everolimus.

Kinsey, C., Lu, T., Deiss, A., Vuolo, K., Klein, L., Rustandi, R.R., Loughney, J.W., 2022. Determination of lipid content and stability in lipid nanoparticles using ultra high‐performance liquid chromatography in combination with a Corona Charged Aerosol Detector. ELECTROPHORESIS 43, 1091–1100. https://doi.org/10.1002/elps.202100244

Kofink, C., Trainor, N., Mair, B., Wöhrle, S., Wurm, M., Mischerikow, N., Roy, M.J., Bader, G., Greb, P., Garavel, G., Diers, E., McLennan, R., Whitworth, C., Vetma, V., Rumpel, K., Scharnweber, M., Fuchs, J.E., Gerstberger, T., Cui, Y., Gremel, G., Chetta, P., Hopf, S., Budano, N., Rinnenthal, J., Gmaschitz, G., Mayer, M., Koegl, M., Ciulli, A., Weinstabl, H., Farnaby, W., 2022. A selective and orally bioavailable VHL-recruiting PROTAC achieves SMARCA2 degradation in vivo. Nat. Commun. 13, 5969. https://doi.org/10.1038/s41467-022-33430-6

Langer, C., Süss, R., 2021. HPLC-DAD-CAD-based approach for the simultaneous analysis of hydrophobic drugs and lipid compounds in liposomes and for cyclodextrin/drug inclusion complexes. J. Pharm. Biomed. Anal. 201, 114120. https://doi.org/10.1016/j.jpba.2021.114120

Lipinski, C.A., Lombardo, F., Dominy, B.W., Feeney, P.J., 2001. Experimental and computational approaches to estimate solubility and permeability in drug discovery and development settings 1PII of original article: S0169-409X(96)00423-1. The article was originally published in Advanced Drug Delivery Reviews 23 (1997) 3–25. 1. Adv. Drug Deliv. Rev. 46, 3–26. https://doi.org/10.1016/S0169-409X(00)00129-0

Mousli, Y., Brachet, M., Chain, J.L., Ferey, L., 2022. A rapid and quantitative reversed-phase HPLC-DAD/ELSD method for lipids involved in nanoparticle formulations. J. Pharm. Biomed. Anal. 220, 115011. https://doi.org/10.1016/j.jpba.2022.115011

Rossi Sebastiano, M., Doak, B.C., Backlund, M., Poongavanam, V., Over, B., Ermondi, G., Caron, G., Matsson, P., Kihlberg, J., 2018. Impact of Dynamically Exposed Polarity on Permeability and Solubility of Chameleonic Drugs Beyond the Rule of 5. J. Med. Chem. 61, 4189–4202. https://doi.org/10.1021/acs.jmedchem.8b00347

Rossi Sebastiano, M., Garcia Jimenez, D., Vallaro, M., Caron, G., Ermondi, G., 2022. Refinement of Computational Access to Molecular Physicochemical Properties: From Ro5 to bRo5. J. Med. Chem. 65, 12068–12083. https://doi.org/10.1021/acs.jmedchem.2c00774

Schönherr, C., Touchene, S., Wilser, G., Peschka-Süss, R., Francese, G., 2009. Simple and precise detection of lipid compounds present within liposomal formulations using a charged aerosol detector. J. Chromatogr. A 1216, 781–786. https://doi.org/10.1016/j.chroma.2008.11.080

Shaw, L.R., Irwin, W.J., Grattan, T.J., Conway, B.R., 2005. The Effect of Selected Water-Soluble Excipients on the Dissolution of Paracetamol and Ibuprofen. Drug Dev. Ind. Pharm. 31, 515–525. https://doi.org/https://doi.org/10.1080/03639040500215784

Shibata, H., Yomota, C., Okuda, H., 2013. Simultaneous Determination of Polyethylene Glycol-Conjugated Liposome Components by Using Reversed-Phase High-Performance Liquid Chromatography with UV and Evaporative Light Scattering Detection. AAPS PharmSciTech 14, 811–817. https://doi.org/10.1208/s12249-013-9967-8

Veber, D.F., Johnson, S.R., Cheng, H.-Y., Smith, B.R., Ward, K.W., Kopple, K.D., 2002. Molecular Properties That Influence the Oral Bioavailability of Drug Candidates. J. Med. Chem. 45, 2615–2623. https://doi.org/10.1021/jm020017n

Vervoort, N., Daemen, D., Török, G., 2008. Performance evaluation of evaporative light scattering detection and charged aerosol detection in reversed phase liquid chromatography. J. Chromatogr. A 1189, 92–100. https://doi.org/10.1016/j.chroma.2007.10.111

Weber, F., Rahnfeld, L., Luciani, P., 2020. Analytical profiling and stability evaluation of liposomal drug delivery systems: A rapid UHPLC-CAD-based approach for phospholipids in research and quality control. Talanta 220, 121320. https://doi.org/10.1016/j.talanta.2020.121320

Whitty, A., Zhong, M., Viarengo, L., Beglov, D., Hall, D.R., Vajda, S., 2016. Quantifying the chameleonic properties of macrocycles and other high-molecular-weight drugs. Drug Discov. Today 21, 712–717. https://doi.org/10.1016/j.drudis.2016.02.005

Yang, Q., Zu, C., Li, W., Wu, W., Ge, Y., Wang, Lingling, Wang, Li, Li, Y., Zhao, X., 2020. Enhanced Water Solubility and Oral Bioavailability of Paclitaxel Crystal Powders through an Innovative Antisolvent Precipitation Process: Antisolvent Crystallization Using Ionic Liquids as Solvent. Pharmaceutics 12, 1008. https://doi.org/10.3390/pharmaceutics12111008
